# Supplementary material for: Health engagement: a systematic review of tools modifiable for use with vulnerable pregnant women
Source: BMJ Open. 2023 Mar 10;13(3):e065720. doi: 10.1136/bmjopen-2022-065720 (PMC10008331; doi:10.1136/bmjopen-2022-065720)
Supplement: Author's manuscript [file bmjopen-2022-065720.draft_revisions.pdf]

BMJ Open is committed to open peer review. As part of this commitment we make the peer review history of every article we publish publicly available.

When an article is published we post the peer reviewers' comments and the authors' responses online. We also post the versions of the paper that were used during peer review. These are the versions that the peer review comments apply to.

The versions of the paper that follow are the versions that were submitted during the peer review process. They are not the versions of record or the final published versions. They should not be cited or distributed as the published version of this manuscript.

BMJ Open is an open access journal and the full, final, typeset and author-corrected version of record of the manuscript is available on our site with no access controls, subscription charges or pay-per-view fees (http://bmjopen.bmj.com).

If you have any questions on BMJ Open's open peer review process please email info.bmjopen@bmj.com

BMJ Open

Health engagement: A systematic review of tools for use with vulnerable pregnant women

Journal:	*BMJ Open*
Manuscript ID	bmjopen-2022-065720
Article Type:	Original research
Date Submitted by the Author:	17-Jun-2022
Complete List of Authors:	Allen, Jyai; Griffith University, School of Nursing and Midwifery; Monash University School of Public Health and Preventive Medicine,<br>Creedy, Debra; Griffith University, School of Nursing and Midwifery<br>Mills, Kyly; Griffith University Griffith Health, School of Health Sciences and Social Work<br>Gamble, Jenny ; Coventry University Faculty of Health and Life Sciences, School of Nursing, Midwifery and Health
Keywords:	Maternal medicine < OBSTETRICS, PRIMARY CARE, PUBLIC HEALTH, SOCIAL MEDICINE

SCHOLARONE™
Manuscripts

I, the Submitting Author has the right to grant and does grant on behalf of all authors of the Work (as defined in the below author licence), an exclusive licence and/or a non-exclusive licence for contributions from authors who are: i) UK Crown employees; ii) where BMJ has agreed a CC-BY licence shall apply, and/or iii) in accordance with the terms applicable for US Federal Government officers or employees acting as part of their official duties; on a worldwide, perpetual, irrevocable, royalty-free basis to BMJ Publishing Group Ltd ("BMJ") its licensees and where the relevant Journal is co-owned by BMJ to the co-owners of the Journal, to publish the Work in this journal and any other BMJ products and to exploit all rights, as set out in our licence.

The Submitting Author accepts and understands that any supply made under these terms is made by BMJ to the Submitting Author unless you are acting as an employee on behalf of your employer or a postgraduate student of an affiliated institution which is paying any applicable article publishing charge ("APC") for Open Access articles. Where the Submitting Author wishes to make the Work available on an Open Access basis (and intends to pay the relevant APC), the terms of reuse of such Open Access shall be governed by a Creative Commons licence – details of these licences and which Creative Commons licence will apply to this Work are set out in our licence referred to above.

Other than as permitted in any relevant BMJ Author's Self Archiving Policies, I confirm this Work has not been accepted for publication elsewhere, is not being considered for publication elsewhere and does not duplicate material already published. I confirm all authors consent to publication of this Work and authorise the granting of this licence.

For peer review only

SHE systematic review

Title

Health engagement: A systematic review of tools for use with vulnerable pregnant women

Authors

Jyai Danielle Nicole **Allen**[1,2], Debra K. **Creedy**[1], Kyly **Mills**[3], Jenny **Gamble**[4,*].

Affiliations

[1] Transforming Maternity Care Collaborative, School of Nursing and Midwifery, Griffith University, 68 University Drive, Meadowbrook, Queensland, 4131, Australia. @transformingMCC

[2] School of Public Health and Preventative Medicine, Monash University, 553 St Kilda Road, Melbourne, Victoria, 3004, Australia.

[3] School of Health Sciences and Social Work, Griffith University, 1 Parklands Drive Southport, Queensland, 4215, Australia.

[4] School of Nursing, Midwifery and Health, Coventry University, Priory Street, Coventry, CV1 5FB, United Kingdom.

Corresponding author

*Jenny Gamble, School of Nursing, Midwifery and Health, Coventry University, Priory Street, Coventry, CV1 5FB, United Kingdom.

Email: ad7603@coventry.ac.uk

MeSH keywords

Maternity care; Midwifery; Patient participation; Systematic review; Surveys and questionnaires

Word count 5305

For peer review only

SHE systematic review

Abstract

Objective: to examine available health engagement tools suitable to, or modifiable for, vulnerable pregnant populations.

Design: systematic review

Eligibility criteria: original studies of tool development and validation related to health engagement, with abstract available in English, published between 2000-2022, sampling people receiving outpatient healthcare including pregnant women.

Data sources: CINAHL Complete, Medline, EMBASE, and Cochrane Database of Systematic Reviews were searched in November 2019 and repeated in April 2022.

Risk of bias: study quality was independently assessed by two reviewers using a quality appraisal checklist. Tools were also mapped against the Synergistic Health Engagement model which centres on women's engagement with maternity care.

Included studies: nineteen studies were included from Canada, Germany, Italy, Netherlands, Sweden, the United Kingdom, and the United States. Four tools were used with pregnant populations, two tools with vulnerable populations, six tools measured patient-provider relationship, four measured patient activation, and three tools measured both relationship and activation.

Synthesis of results: buy-in was not measured in the maternity tools. While non-maternity tools measured some buy-in elements (self-care, feeling hopeful about treatment), other elements significant for vulnerable patient populations (disclosure of risks, acting on health advice) were rarely measured.

Conclusions and implications: no single tool addressed all relevant constructs. Routine measurement of health engagement for vulnerable pregnant populations requires tool development and testing. Greater attention needs to be given to buy-in, health literacy and cultural safety in the development of maternity care engagement tools.

Registration: PROSPERO database (CRD42020214102).

Strength and limitations:

- Health engagement in maternity care is an emerging concept and terms used in this review may not have captured novel or less-used terms
- Study quality was independently assessed by two reviewers using a quality appraisal checklist
- The theoretical model used to synthesise results was missing two constructs relevant to vulnerable populations: health literacy and cultural safety.

For peer review only - http://bmjopen.bmj.com/site/about/guidelines.xhtml

For peer review only

SHE systematic review

Introduction

Health engagement is the willingness and ability of people to actively participate in their health and navigate healthcare systems.[1] This definition includes two related but separate constructs in relation to health and healthcare: activation and health literacy.[2] Activation includes the motivation and confidence to autonomously manage one's health and healthcare; and has been associated with positive health outcomes in several populations with chronic conditions.[3] Health literacy is the extent to which individuals can "obtain, process and understand basic health information",[4] which impacts their behaviours and outcomes.[5] In psychology and related disciplines, the concept of engagement is often measured by therapeutic alliance between patient/client and provider. Therapeutic alliance refers to a collaborative and emotional bond between patient/client and provider that predicts better psychological outcomes with greater certainty than specific interventions.[6]

A recent systematic review demonstrated that therapeutic alliance is rarely measured in nursing, most commonly in mental health nursing (8 papers).[7] A preliminary literature search by the authors found it has never been measured in midwifery, and there has been only one qualitative study of therapeutic alliance in the context of maternity care.[8] Several systematic reviews demonstrate that engagement interventions can improve patients' knowledge and experience, use of health service, health behaviour and health outcomes.[9] However, there are few theoretical frameworks available to inform research about patient engagement, alongside a lack of validated and specific measurement tools.[10] Furthermore, the extent to which such tools are suitable for vulnerable pregnant populations is under-researched.

Vulnerable pregnant women

The term *vulnerable women* encapsulates those women most at risk for poorer maternal health outcomes. In Australia, these populations include adolescent mothers; Aboriginal and Torres Strait Islander mothers; culturally and linguistically diverse mothers; mothers from lower socio-economic backgrounds and mothers who reside in regional and remote areas. Vulnerable women are more likely to have socially determined risk factors which independently predict poor pregnancy outcomes like low birth weight and/or preterm birth.[11] These factors have been systematically researched and include depression,[12] intimate partner violence,[13] housing instability,[14] environmental chemical exposure including tobacco,[15] illicit drug use,[16] inadequate or excessive gestational weight

For peer review only - http://bmjopen.bmj.com/site/about/guidelines.xhtml

gain,[17] genital tract infection,[18] and late or inadequate use of antenatal care.[19] Importantly, the aforementioned factors are modifiable through access to, and engagement with, high quality maternity care.

In order to achieve optimal benefits, maternity care models need to do more than enable pregnant women to just *turn up* to their maternity appointments; the system needs to facilitate *buy-in*.[20] People buy-in when they make "emotional investment and commitment" to healthcare because they are enabled and believe it is "worthwhile and beneficial."[21] People demonstrate buy-in when they participate in health promoting behaviours (e.g. attend for care, have screening tests); and participate in self-care activities including nutrition and exercise, while minimising harm (e.g., reducing or ceasing substance use).[3] Pregnant women buy-in because they hope and believe that this will improve the health and well-being of themselves and their babies.[11] Furthermore, patient buy-in is predicated on trust in health providers' advice and guidance.[22]

There is a clear need to measure health engagement to better understand and address the poorer maternal health outcomes of pregnant women from vulnerable groups. However, we were unable to locate any reviews of health engagement tools for use with pregnant women. Many available health engagement tools have been developed with inpatients who have chronic health conditions, which may not translate to healthy pregnant women receiving outpatient primary care. This gap highlights the need to critically review existing tools to determine their applicability for use with vulnerable pregnant women.

Objectives

This review aimed to (1) describe the nature of health engagement tools used in outpatient populations with or without chronic health conditions; (2) evaluate the reliability and validity of those tools; and (3) use a health engagement framework to evaluate which tools, or components of tools, could be modified for use with vulnerable pregnant populations.

Methods

The systematic review protocol was registered with PROSPERO (number CRD42020214102).

For peer review only - http://bmjopen.bmj.com/site/about/guidelines.xhtml

Theoretical framework

This review was informed by elements of Synergistic Health Engagement (SHE) - an empirical model based on integrated findings from mixed methods research with pregnant adolescents accessing caseload midwifery.[11] Caseload midwifery provides continuity through one-to-one, relationship-based care during pregnancy, birth, and until the first six weeks.[23] According to the SHE model, the first three constructs create the conditions for *buy-in to maternity care* (the fourth construct) which modifies *socially determined predictors of perinatal morbidity* (the fifth construct). The five constructs and their elements are listed below:

1) **Optimal model of care**: continuity of midwifery carer, with 24-hour telephone availability, with community-based, flexible visits
2) **Midwife's attributes:** the skills and personal qualities that enable her to be empowering, trustworthy, and empathetic.
3) **Philosophy / best practice:** the midwife's use of health promotion, woman-centred care, shared decision-making.
4) **Woman's buy-in:** having hope and belief that maternity care will be beneficial, disclosing risk factors, trusting and acting on health advice, and engaging in self-care.
5) **Modifiable social risks for perinatal morbidity**: inadequate antenatal care; smoking, alcohol, or drug use; poor mental health; family violence; inappropriate gestational weight gain; and genito-urinary infection.[20]

The SHE model explains the mechanism by which caseload midwifery reduces the likelihood of poor pregnancy outcomes for vulnerable women.[20] Therefore, SHE was a suitable framework to assess the construct of tools to measure health engagement in maternity care. Application of the SHE model informed the selection of search terms and interpretation of findings.

Eligibility criteria

Inclusion criteria: primary research with study populations of adolescents or adults; and used structured tools to assess healthcare engagement or a related construct (including patient activation, patient-professional relationship).

Exclusion criteria: primary research population inpatients, children or elderly (mean age 65 years or older); engagement not related to receiving healthcare (e.g., technology, research, policy, patient safety); participation not related to healthcare (e.g., work or community

For peer review only - http://bmjopen.bmj.com/site/about/guidelines.xhtml

participation); ineligible study design (pilot studies, intervention studies, qualitative studies, case studies, systematic reviews); earlier iterations of tools which had since been revised or validation of translated tools; shared decision-making tools; tools focussed on a specific health condition.

Search strategy and information sources

The search was conducted in November 2019 and repeated in April 2022. We searched Cumulative Index of Nursing and Allied Health Literature (CINAHL) Complete, Medline, and EMBASE with the following strategy. Key terms for *engagement* were searched as subject terms (patient activation, patient empowerment, patient engagement, patient involvement, patient participation) with the Boolean operator OR. Key terms for *validated tools* were searched in titles (tool, index, measure, scale, instrument, questionnaire). Both searches were combined with Boolean operator AND. For the repeated search, limits were set by date range (2000 – 2022), peer-reviewed, age groups 13 – 44 years (childbearing age). For CINAHL Complete, the search was limited to publication type of questionnaire/scale. A separate search strategy for PubMed used Medical Subject Heading (MeSH) term *patient participation* AND MeSH major heading *surveys and questionnaires.*

Selection and data collection process

We reviewed articles identified by these searches and relevant references found within those articles. When we identified multiple eligible papers from the same study, we included the main paper reporting validity and reliability of the tool. For every eligible study, we extracted data on study design, setting, sample size, population type, age range, research design, name of tool, and psychometric properties of the tool.

Study risk of bias assessment

To our knowledge, there is no suitable tool for assessment of health engagement tools for this population. Therefore, two reviewers independently assessed the quality of included studies using a study-specific tool. We adapted the COSMIN Risk of Bias checklist and updated criteria for measurement properties (Mokkink et al., 2018). Our eleven items for measurement properties included: clear purpose, theoretical constructs, content validity, pilot test, structural validity, internal consistency, reliability, as well as criterion, convergent and predictive validity. Each item was scored according to 1 = adequate or 0 = doubtful/inadequate. Scores were summed with scores of 9 - 11 = high quality; 5 – 8 = moderate quality, and < 5 = poor quality. We assessed all studies for overall quality using the

For peer review only - http://bmjopen.bmj.com/site/about/guidelines.xhtml

STROBE Statement checklist. We investigated the theoretical basis of included tools and compared identified constructs with elements of the SHE model to evaluate utility of tools for use with vulnerable pregnant populations. Ethical approval was not required for this review.

Patient and public involvement

None

Results

Study selection

Figure 1 reports the number of studies obtained, screened, and reviewed. Two authors read 47 full-text articles to further determine eligibility and resolve any disagreement about inclusion by discussion and consensus. Systematic reviews were screened, and where indicated, read in full to search for additional tools, but systematic reviews were not themselves included in this study. Figure 1 indicates reasons for excluding 28 articles from the systematic review following close reading.

Study characteristics

Nineteen tools related to health engagement from 19 studies published between 2000 – 2022 were included. Table 1 summarises the key characteristics of the 19 tools included in the review. Studies were conducted in Canada,[24-27], Germany,[28, 29] Italy,[30, 31] the Netherlands,[32] Sweden,[33] the United Kingdom,[34, 35] and the United States[36-42]. Participants were men and women who were 11 years or older; mean age of participants in each of the included studies was <65 years of age. Four tools were tested with the pregnant population.[24-26, 42] Of these, two tools were co-designed with vulnerable pregnant women (e.g., refugee, socially disadvantaged),[24, 26] and one study adapted a pre-existing tool (Interpersonal Care Processes) to suit a pregnant population (Prenatal Interpersonal Care Processes).[42]

Fifteen studies included non-pregnant participants. Of these, two studies focussed on engagement with mental health services,[33, 34] one focussed on engagement with child protective services,[41]and one solely recruited adolescents.[36] The remaining 11 studies, focussed on health engagement in the general adult population with or without chronic conditions, sampled through online or mailed surveys,[29, 39] in hospital outpatient clinics[28, 30, 31, 33, 37, 38, 40] and general practitioner surgeries.[32, 35] Three studies used the following conceptual frameworks to design their health engagement instruments; the

For peer review only - http://bmjopen.bmj.com/site/about/guidelines.xhtml

Patient Health Engagement Model (PHE-Scale),[31] a conceptual model of outcomes influenced by primary care (PCOQ-24),[35] and therapeutic alliance (KAS-R).[37]

Risk of bias in studies

All tools were valid and reliable with at least adequate quality rated on the 11-point scale. Twelve tools were assessed as high quality (scores 9-11), seven tools were assessed as fair (scores 5-8). The difference in quality related to the extent and adequacy of testing (Table 1).

Results of individual studies

The scales, subscales, and items of the 19 included tools were reviewed and mapped against the SHE model constructs (optimal model of care, midwife attributes, best practice principles buy-in) and related elements. No tool measured all components, and one tool[31] measured none of them.

For peer review only

For peer review only - http://bmjopen.bmj.com/site/about/guidelines.xhtml

Table 1: Characteristics and quality of included tools

Year Country	Tool and aim	Population	Study design	Quality score
**Tools designed for pregnant population accessing maternity care**				
2017[24] *Canada*	**Mothers Autonomy in Decision Making (MADM) scale.** To develop and test a scale to assess women's autonomy and role in decision making in pregnancy	1,672 women with a single provider during pregnancy	Literature review to generate new items and adapt previous items, modification through survey, focus groups and expert panel review. Four working groups refined content with immigrant and refugee women, formerly incarcerated women, women facing socio-economic barriers, and urban/rural settings. Pilot testing with target population with minor rewording.	**9**
2017[26] *Canada*	**Mothers on Respect (MoR) index.** To assess women's experiences with maternity care, including disrespect and discrimination			
2014[25] *Canada*	**Quality of Prenatal Care Questionnaire (QPCQ).** Develop an instrument to measure the quality of prenatal care	422 postpartum women (preliminary) 422 postpartum women (final)	Preliminary tool developed following interviews with pregnant women and health providers, review of prenatal care guidelines, assessment of content validity and rating of importance of items. Preliminary testing and exploratory factor analysis to generate and test final version with separate sample of participants.	**10**
2004[42] *US*	**Prenatal Interpersonal Processes of Care (PIPC).** Develop a reliable and valid multidimensional measure of prenatal interpersonal processes of care for use with ethnically diverse women	363 African American, Latino, Caucasian pregnant women (low-income)	Adaptation of the Interpersonal Processes of Care tool. Focus groups were used to test face validity of items prior to telephone survey with the 30-item tool.	**10**
**Tools designed for non-pregnant population accessing healthcare**				
2021[27] *Canada*	**CADICEE.** To co-construct a tool for measuring degree of partnership between patients and healthcare providers.	206 patients and 38 relatives present during consultation	Patients co-constructed tool. Qualitative analysis of patient experience performed by research team. Patient research advisory group involved in entire construction of tool process. Tool tested for construct and convergent validity and internal consistency.	**7**
2019[30] *Italy*	**Patient-Professional Interaction Questionnaire (PPIQ)**. To investigate how patient evaluate provision of patient-centre care by healthcare professionals and psychometrically test a questionnaire.	1139 inpatients and outpatients	A self-assessment of professionals' provision of PCC was adapted into a patient-rated form. The questionnaire structure, reliability, susceptibility to social desirability, and associations with other variables were tested.	**8**
2019[29] *Germany*	**Patient Enablement (PEN-13).** To develop and psychometrically test a German-language survey instrument that measures patient enablement generically and in greater detail than previous instruments	354 adults registered with the integrated care system	Analysis of key concepts from the literature by a multidisciplinary team informed item development. Construct and structural validity, and internal consistency were tested.	**9**
2018[35] *UK*	**Primary Care Outcomes Questionnaire (PCOQ).** To test the PCOQ which aims to capture a broad range of outcomes relevant to primary care.	602 adult general practitioner patients with and without chronic conditions	Questionnaire developed through use of a conceptual model, interviews with patients and clinicians, expert review through Delphi study, and refinement of tool through cognitive interviews prior to psychometric testing.	**10**

For peer review only - http://bmjopen.bmj.com/site/about/guidelines.xhtml

2015[31] *Italy*	**Patient Health Engagement Scale (PHE-Scale).** To evaluate the psychometric properties of the PHE-scale and to evaluate the association between PHE-scale scores and concurrent measures.	382 adults with chronic disease	Items based on previous conceptualization of patient engagement (the PHE-model). Items based on a systematic analysis of the literature and an extensive qualitative study. Tool was pilot-tested and validated using confirmatory factor analysis.	**10**
2015[39] *US*	**Altarum Consumer Engagement (ACE) measure.** To assess an individual's engagement in health and healthcare decisions.	2,079 adults through online survey	Item generation through literature review, piloting and refinement of items. Testing of tool through web-based survey	**9**
2014[36] *US*	**Youth Engagement with Health Services (YEHS!).** To create and validate a survey instrument designed to measure youth engagement with health services	354 ethnically diverse high school students, school health centres	Item generation through literature review including existing validated measures, expert opinion, and interviews with adolescents. Instrument pilot tested and refined.	**8**
2010[28] *Germany*	**Questionnaire on the Quality of Physician-Patient Interaction (QQPPI).** To assess the quality of physician-patient interactions.	147 adults and 19 physicians in outpatient clinic	Item generation from exploratory in-depth interviews with 20 patients. Literature review used to screen for additional items. An expert panel reviewed the final 14-item questionnaire.	**10**
2008[33] *Sweden*	**Health Promotion Intervention Questionnaire (HPIQ).** To measure patients' subjective experiences of health promotion interventions in mental health services.	135 adults who accessed a mental health service	Item generation informed by qualitative research; cross-section survey used to pilot test tool.	**7**
2008[37] *US*	**Kim Alliance Scale – Revised (KAS-R).** To measure quality of the therapeutic alliance from patient's perspective, including patient empowerment.	601 participants from two outpatient clinics	Conceptual framework developed through literature review and qualitative research. Original 48-item KAS tool piloted as paper-based survey and refined to 30-items. KAS tool further refined to 16-items through exploratory and validation studies.	**10**
2005[40] *US*	**Patient Activation Measure (PAM-13).** To conceptualize and measure "activation" in patients and consumers	1,515 adults with or without a chronic condition	Mixed methods study including national expert consensus and patient focus groups to define and identify domains. Pilot testing and refinement prior to national survey.	**10**
2005[41] *US*	**Client Engagement in Child Protective Services.** To develop and test a multidimensional measure of client engagement in child welfare services.	287 primary caregivers with an open child protective services case	Five dimensions of engagement based on literature review, interviews with child welfare workers and clients. Three expert panels provided feedback on items, validity, and format. Construct validity and internal consistency were tested.	**8**
2004[34] *UK*	**Engagement measure (self-report version).** To develop a reliable self-report measure of engagement based on Hall, Meaden, Smith, & Jones 2001 observer-rated measure of engagement	25 unemployed adults accessing outreach mental health services	Tool developed based on an observer-rated measure of engagement. The *process* of developing the altered self-report measure of engagement not provided. Construct validity, including factor analysis were not assessed.	**6**
2004[32] *Holland*	**Patient-Doctor Relationship Questionnaire (PDRQ-9).** To develop and validate a questionnaire to assess the patient-doctor relationship	110 general practice patients, 55 epilepsy clinic patients	The HAQ of Luborsky basis for item creation. Pilot testing (n = 8) resulted in the additional item. Factor analysis, test re-test reliability and internal consistency were undertaken.	**6**
2001[38] *US*	**Facilitation of Patient Involvement (FPI) scale** To measure the degree to which patients perceive their physicians actively facilitate or encourage involvement in their own healthcare.	236 adults (pilot) 338 adults (survey)	Item generation through literature review and revision based on expert feedback. Pilot study of 9-item scale using 6-point Likert responses.	**10**

For peer review only - http://bmjopen.bmj.com/site/about/guidelines.xhtml

Health engagement tools for pregnant women

Four tools were designed to measure health engagement in maternity care. Both the MADM tool[24] and MoR index[26] measured shared decision-making. In addition, the MoR index[26] measured providers attributes associated with (dis)respectful care. While the MoR index[26] measured the unwillingness of pregnant women to ask questions or share concerns with their provider, it did not specifically measure disclosure of risks, which is a significant element of buy-in. The 2014 QPCQ[25] included six subscales: 1) information sharing, 2) anticipatory guidance, 3) sufficient time, 4) approachability, 5) availability, 6) support and respect. The QPCQ[25] subscales mapped against optimal model of care, provider attributes, and best practice principles including health promotion; but did not measure buy-in. The 2004 PIPC[42] included three dimensions (subscales): 1) communication (elicitation of, and responsiveness to patient; explanations of processes of care; empowerment/self-care); 2) patient-centred decision making; and 3) interpersonal style (friendliness and courteousness; respectfulness/emotional support; lack of perceived discrimination). The PIPC[42] measured provider attributes including being perceived as discriminatory, empowering, and empathetic. The PIPC[42] measured elements of best practice including woman-centred care and shared decision-making. While the PIPC[42] was developed with an ethnically diverse, low-income sample of pregnant women, it does not measure buy-in.

Health engagement tools for vulnerable people

Two tools were designed for people who were vulnerable due to age (adolescents), or engagement with child protective services. The Youth Engagement in Health Services (YEHS!)[36] survey measured adolescent health engagement across four components: 1) receipt of anticipatory health guidance, 2) experience of care, 3) health access literacy, and 4) health self-efficacy. Health topics included in the anticipatory guidance component are relevant to health promotion during pregnancy; and could be easily adapted in future tool development. Yatchmenoff (2005) developed a 19-item client-centred health engagement model with four factors: 1) buy-in, 2) receptivity, 3) working relationship, and 4) mistrust.[41] The factors, and items, in this tool are relevant to buy-in and receptivity of vulnerable pregnant populations and the midwife-woman relationship.

General health activation tools

The Patient Activation Measure (PAM-13)[40] was the first tool to define the construct of patient activation, and is the most widely used measure of patient engagement. The 13 items focus on the patient’s perceived knowledge, confidence and ability to manage their health

For peer review only - http://bmjopen.bmj.com/site/about/guidelines.xhtml

condition.[40] While commonly used, the tool has limited generalisability to vulnerable populations because of readability for people with low health literacy.[43] Furthermore, because activation is constructed solely in terms of *confidence* and *ability*, this obfuscates significant barriers to health engagement that occur through social determinants of health and poor patient-provider relationships.

The Patient Enablement (PEN-13) questionnaire (German version) focusses specifically on measuring skills patients without chronic conditions had to manage their health care.[29] Like the PAM-13,[40] this tool measures an aspect of patient engagement without the context of the patient-provider relationship, which limits usefulness for modification for a maternity tool.

The Patient Health Engagement (PHE) scale[31] focusses on patients who receive a health diagnosis, and measures items that occur prior to, and predict, the individual's ability to engage in health care. For example, *When I think about my disease: I feel blackout, I am in alarm, I am aware, I feel positive.*[31] For both reasons, the PHE scale[31] is not relevant to maternity health engagement.

The Altarum Consumer Engagement (ACE) measure[39] included 20-items evenly divided across four subscales: 1) commitment, 2) informed choice, 3) navigation, and 4) ownership. The ACE[39] measures individuals' confidence to navigate the healthcare system and take responsibility for their health. Only one item refers to interaction with a health professional (i.e., *I feel comfortable talking to my doctor about my health*). [39]

Patient-provider relationship tools

Six tools focussed on health engagement through the lens of the patient-provider relationship. The Kim Alliance Scale Revised (KAS-R)[37] was developed to measure *therapeutic alliance* in healthcare. The KAS-R[37] included 16 items evenly divided across four subscales: 1) collaboration, 2) integration, 3) empowerment, and 4) communication. Items which measured mutual goals and respect are useful to health engagement for pregnant women. The wording of some items (e.g., *I am allowed in the decision-making process*)[37] reflects that this tool was developed over 15-years ago, prior to significant progress in the area of shared decision-making. More recently published tools, like the MADM tool,[24] have more appropriate wording and description of decision-making in maternity care.

The Patient Doctor Relationship Questionnaire (PDRQ-9)[32] contained nine short and general, and potentially vague, statements about satisfaction with relationship with the

For peer review only - http://bmjopen.bmj.com/site/about/guidelines.xhtml

primary care provider (e.g., *My primary care provider understands me*). In comparison, the Questionnaire on Quality of Physician-Patient Interactions (QQPPI)[28] contained 14 statements which were more specific (e.g., *The physician seemed genuinely interested in my problems*). Likewise, the Facilitation of Patient Involvement (FPI) scale[38] asked patients to rate how often their physician does nine things. For example, *My doctor explains all the treatment options to me so that I can make an informed choice.*[38] The Patient-Professional Interaction Questionnaire (PPIQ)[30] included 16 items, of which several measured whether the professional was interested in (e.g. *what I know about my disease/prognosis, what I want from care*). The Health Promotion Intervention Questionnaire (HPIQ)[33] measures patient experiences of health promotion as received through their relationship with a key worker. The HPIQ[33] consists of 19-items across four factors: Alliance, Empowerment, Educational Support, and Practical Support. The items are commonly superficially worded (e.g., *Key worker treats me in a friendly way and often smiles*) which does not measure the therapeutic nature of the patient-provider relationship.

Comprehensive health engagement tools

Three tools measured both patient-provider relationship and components of patient activation. The CADICEE tool[27] comprehensively addresses the complex components of health engagement using 24 items across seven dimensions: 1) relationship of **C**onfidence or trust between patients and health care providers, 2) patient **A**utonomy, 3) patient participation in **D**ecisions related to care, 4) shared **I**nformation on patient health status and care, 5) patient personal **C**ontext, 6) **E**mpathy, and 7) recognition of **E**xpertise.[27]

The PCOQ-24[35] has 24-items across four dimensions: 1) health and wellbeing, 2) health knowledge and self-care, 3) confidence in health provision, and 4) confidence in health plan. Items ask about the skills and attributes of *the doctors or nurses you usually see* as well as patient knowledge and ability to self-manage their health condition.[35]

The Engagement Measure (client version)[34] has six key areas: 1) appointment keeping, 2) client-keyworker relationship, 3) communication/openness with key workers, 4) usefulness of treatment, 5) involvement with treatment, and 6) taking medication. The professional's attributes were not described (*e.g., How well do you get on with _______?*) which makes items so general that their value is limited.

For peer review only - http://bmjopen.bmj.com/site/about/guidelines.xhtml

Synthesis of Results

The results from all studies were synthesised through mapping against SHE model constructs of model of care; health professional's skills and attributes; best practice; and buy-in.

Model of care

The availability and flexibility of the health professional to meet the individual's needs, were largely measured in terms of having sufficient time and being contactable. For example, the PIPC item: *How often did providers give you enough time to say what you thought was important?*[42] The QPCQ[25] differentiated between being able to contact *someone* versus *my provider* when concerns arose. Four general health engagement tools (QQPPI[28], PPIQ[30], PDRQ-9[32], YEHS![36]) included items about having *enough time* with a health professional. Only one general tool (PDRQ-9[32]) measured accessibility: *I find my primary care provider easily accessible.*

Health professional's skills and attributes

Most tools include subscales or items that measured whether the individual perceived their provider as empowering, trustworthy, or empathetic; or provider actions that could be interpreted to demonstrate these qualities. Of the four maternity tools, two tools included the terms 'doctor or midwife' and asked respondents to indicate which healthcare provider their responses referred to.[24, 26] Whereas the QPCQ referred generally to a *prenatal care provider,*[25] and the PIPC measured experiences about *doctors, nurses and other providers*.[42]

Empowering

An empowering health professional refers to someone who uses communication skills to effectively share information with pregnant women. Any items that were specifically about how the health professional facilitated shared decision-making were mapped against that element of best practice. Two maternity tools measured attributes related to whether the health professional was empowering. For example, the QPCQ item: *I fully understood the reasons for blood work and other tests that my prenatal care provider(s) ordered for me*[25] and the PIPC item: *How often did providers tell you what they were doing as they gave you a physical examination?*[42]

For peer review only - http://bmjopen.bmj.com/site/about/guidelines.xhtml

Most non-maternity tools included at least one item that measured whether the health professional acted in ways that were empowering (QQPPI[28], PPIQ[30], PCOQ[35], CADICEE[27], PAM-13[40], KAS-R[37], FPI[38], PEN-13[29], YEHS![36]). The KAS-R[37] included an Empowerment subscale with items such as: *I have an active partnership with my provider*. Whereas the FPI[38] included a negatively worded item: *My doctor discourages my questions.*

Trustworthy

Three maternity tools (QPCQ[25], PIPC[42], MoR[26]) measured outcomes related to perceptions of the health professional being trustworthy, including perceived discrimination, support, and respect. However, only two tools specifically measured racism. For example, the PIPC item: *How often did you feel discriminated against because of your race or ethnicity?* [42] Whereas the MoR included multiple items related to disrespectful care including *When I had my baby I felt that I was treated poorly by my (midwife, doctor): Because of my race, ethnicity, cultural background or language.[26]*

A third of the non-maternity tools (YEHS![36], CECPS[41], CADICEE[27], PCOQ[35], PDRQ-9[32]) measured patient perceptions of patient-provided relationship in terms of trust. For example, the YEHS! item: *I have a safe and trusting relationship with at least one doctor or healthcare provider.*[36] Whereas the PCOQ-24[35] included a more generic question regarding doctors and nurses in the general practice: *You can trust them.* This item does not speak to a patient-provider relationship, nor continuity of care with a specific provider, so it of limited utility.

Empathetic

Both the QPCQ[25] and PIPC[42] have sub-scales related to empathy including demonstrating interest in the woman as a person and communicating emotional support. For example, the QPCQ item: *My prenatal care provider was interested in how my pregnancy was affecting my life*[25] and the PIPC item: *How often did providers help you feel less worried about your pregnancy?* [42]

About half of the general tools (YEHS![36], QQPPI[28], CADICEE[27], PPIQ[30], KAS-R[37], HPIQ[33]) measured patient-provider relationship in terms of empathy. Similarly, to the PIPC item, a QQPPI item measured the ability of the provider to reassure the patient: *The physician did all he/she could to put me at ease.*[28] Whereas some tools more explicitly

For peer review only - http://bmjopen.bmj.com/site/about/guidelines.xhtml

measured the emotional element of the patient-provider relationship. For example, the PPIQ item: *He/she was able to put him/herself in "my shoes"*[30]

Best practice principles

In the SHE model, health promotion, woman-centred care and shared decision-making were captured as a construct titled *philosophical commitments*, in other words, best practice principles.

Health promotion

Health promotion in maternity care includes discussion and recommendations around diet, exercise, emotional health, interpersonal relationships, social support, harm minimisation for smoking, drugs and alcohol.[20] Two included maternity tools measured constructs directly related to promotion of healthy behaviours. A QPCQ subscale measured whether women were provided with education about components of health including diet, exercise, alcohol, emotional health; for example: *I was given adequate information about depression in pregnancy*.[25] In contrast, a PIPC sub-scale asked more broadly about whether pregnant women had received lifestyle advice.[42] The PIPC also included an item about the health professional's role in promoting the woman to engage in healthy behaviours: *How often did providers tell you what you could do to take care of yourself and your pregnancy at home?* [42]

Few non-maternity tools included items around health promotion. However, both PAM-13[40] and ACE[39] included several items around patient responsibility in self-care and healthy behaviours. Whereas the HPIQ, designed for participants with mental health concerns, includes the item: *My key worker informs me about what I need in order to feel better*. [33] The YEHS! was the most relevant because it provided a list of specific educational topics beyond diet and exercise, relevant to vulnerable populations:

> *In the* ***last 12 months****, did a doctor or other health care provider talk with you about the following: weight, healthy eating or diet, physical activity or exercise, your emotions or moods, how you deal with stress, sleep, sexual risk reduction (STDs) [36]*

Woman-centred / individualised care

Most maternity tools measured whether the individual felt care was individualised to their specific values, preferences, and desires (QPCQ[25], MoR[26], PIPC[42]). For example, the QPCQ item: *I was supported by my prenatal care provider(s) in doing what I felt was right*

For peer review only - http://bmjopen.bmj.com/site/about/guidelines.xhtml

for me.[25] Only three non-maternity tools assessed whether the provider communicated interest in what the patient knew and wanted from their care (QQPPI[28], CADICEE[27], PPIQ[30]). For example, the CADICEE tool measured a further element of individualisation around patient expertise about themselves and their health: *Did you feel that this professional recognized your expertise and took it into account?*[27]

Shared decision-making

Shared decision-making was measured in all four maternity tools. The most comprehensive measures were included in the MADM tool[24] and MoR index[26] which were codesigned with vulnerable pregnant women. Example items include: *My doctor or midwife explained the advantages and disadvantages of the maternity care options* (MADM[24]) and *Overall while making decision during my pregnancy I felt coerced into accepting the options my (midwife, doctor) recommended* (MoR[26]). About half of the non-maternity tools included items that measured shared decision-making (QQPPI[28],CADICEE[27], PPIQ[30], KAS-R[37], FPI[38], HPIQ[33]). For example, the QQPPI item: *The physician and I made treatment decisions together.*[28]

Buy-in

Buy-in was not measured by the included maternity tools, whereas each element (self-care, hope/belief, disclosure of risk, and accepting help) was measured by some of the non-maternity tools.

Self-care

Several non-maternity tools (PAM-13[40], ACE[39], PCOQ[35], PEN-13[29]) measured the individuals' confidence and willingness to manage their health including knowing when and how to access help, following through on plans and goals, maintaining a healthy lifestyle, and preventing deterioration of their health. For example, the PAM-13 item *I am confident that I can take actions that will help prevent or minimise some symptoms or problems associated with my health condition*.[40] These items were generally worded around a diagnosed health condition, which does not translate easily to pregnancy. The PCOQ-24[35] included a more general question around self-care and health: *Thinking about your level of knowledge, how much do you know how best to look after yourself and stay healthy.*

Hope and belief

An individual's mindset, their interpretation of whether healthcare is "worth it" or not, is a motivating factor in health engagement. This element was measured by a third of the non-

For peer review only - http://bmjopen.bmj.com/site/about/guidelines.xhtml

maternity tools (CECPS[41], PCOQ[35], PPIQ[30], EM[34], HPIQ[33]), commonly in a single item. The CECPS[41] included five items that measured mutual goals and optimism for a better future through engagement with child protective services. For example, *Working with ______ has given me more hope about how my life is going to go in the future*.[41]

Disclosure

The element of disclosure of risk factors (e.g., family violence) was not specifically measured by any of the 19 included tools. Of the four maternity tools, only the MoR index[26] included three items regarding whether women perceived they were held back from asking questions or discussing concerns. About half of the non-maternity tools (YEHS!, CECPS[41], QQPPI[28], CADICEE[27], EM, PAM-13[40], PEN-13[29]) included items related to disclosure. For example, whether individuals could share problems or concerns without being directly asked by the provider (*I have no difficulty in telling my doctor about my concerns or fears, even if he or she does not address them directly* – PEN-13[29]). Whereas the CECPS includes an item about non-disclosure (*Anything I say they're going to turn it around and make me look bad*).[41]

Accepting help, referral, treatment

Acceptance of help, including agreeing to referrals and following up with tests, community resources and support from other providers was not measured. Some maternity tools measured whether information was provided about screening tests.[42] Providing an explanation is empowering, and may increase the likelihood of women doing the test, but it does not measure buy-in. Agreeing to treatment, going along with treatment, and wanting help were measured by non-maternity tools. A third of the non-maternity tools (YEHS![36], CECPS[41], PCOQ[35], EM[34], PAM-13[40]) measured willingness to accept or act on help offered by the care provider. For example, *When I make a plan with a doctor or other health care provider, I can follow through on the plan at home* in the YEHS![36] The PCOQ-24 include items with greater specificity around follow through, for example: *How much of your doctors or nurses advice are you following on: your medication or treatment; leading a healthy lifestyle.[35]*

Discussion

This is the first systematic review to evaluate the characteristics, quality, and sufficiency of tools to measure health engagement in vulnerable pregnant populations. Twelve tools were high quality and validated for use with generally healthy adolescents and adults in high-

For peer review only - http://bmjopen.bmj.com/site/about/guidelines.xhtml

income healthcare settings. The SHE framework was useful to map constructs to potential scales, subscales, and items. Buy-in was not measured in the included maternity tools, and only partially measured in some of the general patient engagement tools. Specifically, disclosure and accepting help were rarely measured.

The review was limited to studies with abstracts available in English. There may be studies in different languages that were not located or excluded from review. While we did review tools used with other populations, our focus on concepts of care relevant to pregnant women may have been limiting. However, registration of the review protocol confirmed the relative lack of attention health engagement generally and with pregnant women specifically. Although, application of the SHE model as a framework for the review ensured that a broad range of relevant concepts were considered, two elements (health literacy and cultural safety) were identified as missing. Inclusion of these elements from the outset may have influenced the search terms and outcomes. Furthermore, the SHE model has been tested only with adolescent mothers.

Health literacy

Elements of health literacy were broadly measured including how well participants could understand instructions or explanations provided to them by care providers (KAS-R[37], QQPPI[28]); or how users sought and used information (ACE[39]). Interestingly, the provider's knowledge and skills around maternal health literacy were not measured in the included maternity engagement tools. A national survey reported that most Australian midwives have not received education about health literacy, do not formally assess it, and either never or only sometimes use techniques to facilitate it.[44] There are currently no rapid measures of maternal health literacy available.[45]

Cultural safety

Racism undermines equitable access to services and predicts poor maternal and perinatal outcomes.[46] Internationally, First Nations women experience racism, prejudice and discrimination, and lack of respect for cultural practices in maternity care.[47, 48] Indeed, social risk factors are exacerbated by health care that is perceived as racist or culturally unsafe; because it contributes to reduced adherence to health advice or complete disengagement from services.[49] Women who do not wish to attend, or are prevented from attending, antenatal care are less likely to receive advice, screening and reassurance about the progress of pregnancy. Beyond attendance, cultural safety is central to women's decision to

For peer review only - http://bmjopen.bmj.com/site/about/guidelines.xhtml

disclose risk factors, like domestic violence, to their care providers.[50] Perceived lack of cultural safety through hospital-based, fragmented care is a barrier to attendance, whereas culturally safe, midwifery continuity of care in the community is facilitative.[51]A recent study of the impact of culturally safe maternity care, concluded that synergistic engagement is the mechanism by which these innovative services effectively improve maternal and perinatal outcomes for First Nations women in Australia.[52] However, in the absence of a tool validated to measure SHE constructs, this explanation remains theoretical.

Antenatal attendance

The term *engagement* is commonly used as a synonym for *attendance* in the research literature. For example, a recent scoping review on improving antenatal engagement for Aboriginal women used measures of non-attendance, less attendance, and late attendance.[53] While antenatal attendance is important, without *buy-in* to maternity care and resultant modification of social risk factors, its' efficacy is limited. The ability to determine appropriate medical and/or psychosocial intervention is predicated on women feeling safe to disclose risks to their maternity care providers.[54] Future studies of maternity care engagement should include specific measures that can assess not only the quantity of engagement (attendance) but quality.

Relationship-based care

Testing of health promoting interventions, delivered in the absence of relationship-based care, has been proposed to improve perinatal outcomes.[55] While health promotion is an important part of quality maternity care, the provision of motivational counselling around social risk factors, does not necessarily translate into women acting on advice. A trusting, provider-woman relationship is foundational to pregnant women's willingness and ability to engage.[20] Women randomised to receive midwifery continuity, versus fragmented care, characterised their midwife as *empowering* and *'endorphic'* (makes me feel safe, loved, relaxed); compared to simply informative, competent, kind.[56] We recommend interventions designed to promote healthy behaviours during pregnancy are embedded in relationship-based models of care to bolster their efficacy.

Implications for practice and future research

Maternal health engagement is crucial to improving perinatal outcomes for vulnerable groups. Models of maternity care can be designed to enhance or hinder engagement. Therefore, routine measurement of health engagement is an important activity to monitor and

For peer review only - http://bmjopen.bmj.com/site/about/guidelines.xhtml

improve the quality of maternity care for vulnerable pregnant women. While 12 tools were deemed to be high quality, no single tool addressed all relevant constructs. Development and testing of a tool that addresses these concepts within a revised *Synergistic Health Engagement* model is required.

For peer review only

For peer review only - http://bmjopen.bmj.com/site/about/guidelines.xhtml

Competing interests' statement: The authors declare no competing interests.

Funding statement: This research received no specific grant from any funding agency public, commercial or not-for-profit.

Author contributions: Author 1: conceived and designed the analysis, collected the data, analysed the results, wrote the paper. **Author 2:** conceived and design the analysis, performed quality appraisal of included articles, revised the paper. **Author 3:** performed quality appraisal of included articles, revised the paper. **Author 4:** conceived and designed the analysis, revised the paper.

Ethical approval statement: This study did not involve animal or human participants; therefore, ethical approval was not required.

Data sharing statement: Data sharing not applicable as no datasets generated and/or analysed for this study.

For peer review only

For peer review only - http://bmjopen.bmj.com/site/about/guidelines.xhtml

SHE systematic review

References

1. Friis K, Lasgaard M, Osborne RH, Maindal HT. Gaps in understanding health and engagement with healthcare providers across common long-term conditions: A population survey of health literacy in 29 473 Danish citizens. British Medical Journal Open. 2016;6(1):e009627.
2. Smith SG, Curtis LM, Wardle J, von Wagner C, Wolf MS. Skill set or mind set? Associations between health literacy, patient activation and health. PloS one. 2013;8:e74373.
3. Graffigna G, Barello S, Bonanomi A. The role of Patient Health Engagement Model (PHE-model) in affecting patient activation and medication adherence: A structural equation model. PloS one. 2017;12:e0179865.
4. Ratzan SC. Health literacy: Communication for the public good. Health Promotion International. 2001;16(2):207-14.
5. Kilfoyle KA, Vitko M, O'Conor R, Bailey SC. Health literacy and women's reproductive health: A systematic review. Journal of Women's Health. 2016;25(12):1237-55.
6. Martin DJ, Garske JP, Davis MK. Relation of the therapeutic alliance with outcome and other variables: A meta-analytic review. Journal of Consulting and Clinical Psychology. 2000;68(3):438-50.
7. Hartley S, Raphael J, Lovell K, Berry K. Effective nurse-patient relationships in mental health care: A systematic review of interventions to improve the therapeutic alliance. International Journal of Nursing Studies. 2020;102:103490.
8. Doherty ME. Therapeutic alliance: A concept for the childbearing season. J Perinat Educ. 2009;18(3):39-47.
9. Coulter A, Ellins J. Effectiveness of strategies for informing, educating, and involving patients. British Medical Journal 2007;335(7609):24-7.
10. Manafo E, Petermann L, Mason-Lai P, Vandall-Walker V. Patient engagement in Canada: A scoping review of the 'how' and 'what' of patient engagement in health research. Health Research Policy and Systems. 2018;16(1):5.
11. Allen JDN. (How) does the way maternity care is provided affect the health and well-being of young women and their babies? A mixed methods research project [thesis]. Brisbane, Australia: Australian Catholic University; 2015.
12. Accortt EE, Cheadle AC, Dunkel Schetter C. Prenatal depression and adverse birth outcomes: An updated systematic review. Journal of Maternal and Child Health. 2015;19(6):1306-37.
13. Nesari M, Olson JK, Vandermeer B, Slater L, Olson DM. Does a maternal history of abuse before pregnancy affect pregnancy outcomes? A systematic review with meta-analysis. BMC pregnancy and childbirth. 2018;18(1):404.
14. DiTosto JD, Holder K, Soyemi E, Beestrum M, Yee LM. Housing instability and adverse perinatal outcomes: A systematic review. American Journal of Obstetrics & Gynecology MFM. 2021;3(6):100477.
15. Porpora MG, Piacenti I, Scaramuzzino S, Masciullo L, Rech F, Benedetti Panici P. Environmental contaminants exposure and preterm birth: A systematic review. Toxics. 2019;7(1):11.
16. Dos Santos JF, de Melo Bastos Cavalcante C, Barbosa FT, Gitaí DLG, Duzzioni M, Tilelli CQ, et al. Maternal, fetal and neonatal consequences associated with the use of crack cocaine during the gestational period: A systematic review and meta-analysis. Archives of Gynecology and Obstetrics. 2018;298(3):487-503.
17. Goldstein RF, Abell SK, Ranasinha S, Misso M, Boyle JA, Black MH, et al. Association of gestational weight gain with maternal and infant outcomes: A systematic review and meta-analysis. Journal of the American Medical Association. 2017;317(21):2207-25.
18. Sangkomkamhang US, Lumbiganon P, Prasertcharoensuk W, Laopaiboon M. Antenatal lower genital tract infection screening and treatment programs for preventing preterm delivery. Cochrane Database of Systematic Reviews. 2015;2015(2):Cd006178.
19. Feijen-de Jong EI, Jansen DE, Baarveld F, van der Schans CP, Schellevis FG, Reijneveld SA. Determinants of late and/or inadequate use of prenatal healthcare in high-income countries: A systematic review. European Journal of Public Health. 2012;22(6):904-13.

For peer review only - http://bmjopen.bmj.com/site/about/guidelines.xhtml

20. Allen J, Kildea S, Stapleton H. How optimal caseload midwifery can modify predictors for preterm birth in young women: Integrated findings from a mixed methods study. Midwifery. 2016;41:30-8.
21. Gopalan G, Goldstein L, Klingenstein K, Sicher C, Blake C, McKay MM. Engaging families into child mental health treatment: Updates and special considerations. Journal of the Canadian Academy of Child and Adolescent Psychiatry 2010;19(3):182-96.
22. Becker ER, Roblin DW. Translating primary care practice climate into patient activation: The role of patient trust in physician. Medical Care. 2008;46(8):795-805.
23. Sandall J, Soltani H, Gates S, Shennan A, Devane D. Midwife-led continuity models versus other models of care for childbearing women. Cochrane Database of Systematic Reviews. 2016;4:CD004667.
24. Vedam S, Stoll K, Martin K, Rubashkin N, Partridge S, Thordarson D, et al. The Mother's Autonomy in Decision Making (MADM) scale: Patient-led development and psychometric testing of a new instrument to evaluate experience of maternity care. PloS one. 2017;12(2):e0171804.
25. Heaman MI, Sword WA, Akhtar-Danesh N, Bradford A, Tough S, Janssen PA, et al. Quality of prenatal care questionnaire: Instrument development and testing. BMC pregnancy and childbirth. 2014;14:188.
26. Vedam S, Stoll K, Rubashkin Nea. The Mothers on Respect (MOR) index: Measuring quality, safety, and human rights in childbirth. SSM - Population Health. 2017;3:201-10.
27. Pomey MP, Clavel N, Normandin L, Del Grande C, Philip Ghadiri D, Fernandez-McAuley I, et al. Assessing and promoting partnership between patients and health-care professionals: Co-construction of the CADICEE tool for patients and their relatives. Health Expectations. 2021;24(4):1230-41.
28. Bieber C, Müller KG, Nicolai J, Hartmann M, Eich W. How does your doctor talk with you? Preliminary validation of a brief patient self-report questionnaire on the quality of physician-patient interaction. Journal of Clinical Psychology in Medical Settings. 2010;17(2):125-36.
29. Siegel A, Ehmann AT, Meyer I, Gröne O, Niebling W, Martus P, et al. PEN-13: a new generic 13-item questionnaire for measuring patient enablement (German version). International Journal of Environmental Research and Public Health. 2019;16(23):4867.
30. Casu G, Gremigni P, Sommaruga M. The Patient-Professional Interaction Questionnaire (PPIQ) to assess patient centered care from the patient's perspective. Patient Education and Counseling. 2019;102(1):126-33.
31. Graffigna G, Barello S, Bonanomi A, Lozza E. Measuring patient engagement: Development and psychometric properties of the Patient Health Engagement (PHE) Scale. Frontiers in Psychology. 2015;6.
32. Van der Feltz-Cornelis CM, Van Oppen P, Van Marwijk HW, De Beurs E, Van Dyck R. A patient-doctor relationship questionnaire (PDRQ-9) in primary care: Development and psychometric evaluation. General Hospital Psychiatry. 2004;26(2):115-20.
33. Svedberg P, Arvidsson B, Svensson B, Hansson L. Psychometric characteristics of a self-report questionnaire (HPIQ) focusing on health promotion interventions in mental health services. International Journal of Mental Health Nursing. 2008;17(3):171-9.
34. Gillespie M, Smith J, Meaden A, Jones C, Wane J. Clients' engagement with assertive outreach services: A comparison of client and staff perceptions of engagement and its impact on later engagement. Journal of Mental Health. 2004;13(5):439-52.
35. Murphy M, Hollinghurst S, Cowlishaw S, Salisbury C. Primary Care Outcomes Questionnaire: Psychometric testing of a new instrument. British Journal of General Practice. 2018:bjgp18X695765.
36. Sebastian RA, Ramos MM, Stumbo S, McGrath J, Fairbrother G. Measuring youth health engagement: Development of the youth engagement with health services survey. Journal of Adolescent Health. 2014;55(3):334-40.
37. Kim SC, Kim S, Boren D. The quality of therapeutic alliance between patient and provider predicts general satisfaction. Military Medicine. 2008;173(1):85-90.

For peer review only - http://bmjopen.bmj.com/site/about/guidelines.xhtml

38. Martin LR, Robin Di Matteo M, Lepper HS. Facilitation of Patient Involvement in Care: Development and Validation of a Scale. Behavioral medicine (Washington, DC). 2001;27(3):111-20.
39. Duke CC, Lynch WD, Smith B, Winstanley J. Validity of a new patient engagement measure: The Altarum Consumer Engagement (ACE) measure. Patient. 2015;8(6):559-68.
40. Hibbard JH, Stockard J, Mahoney ER, Tusler M. Development of the Patient Activation Measure (PAM): Conceptualizing and measuring activation in patients and consumers. Health Services Research 2005;39(4p1):1005-26.
41. Yatchmenoff DK. Measuring client engagement from the client's perspective in nonvoluntary child protective services. Research on Social Work Practice. 2005;15(2):84-96.
42. Wong ST, Korenbrot CC, Stewart AL. Consumer assessment of the quality of interpersonal processes of prenatal care among ethnically diverse low-income women: Development of a new measure. Womens Health Issues. 2004;14.
43. Wolf MS, Smith SG, Pandit AU, Condon DM, Curtis LM, Griffith J, et al. Development and validation of the Consumer Health Activation Index. Medical Decision Making. 2018;38(3):334-43.
44. Creedy DK, Gamble J, Boorman R, Allen J. Midwives' self-reported knowledge and skills to assess and promote maternal health literacy: A national cross-sectional survey. Women and birth : journal of the Australian College of Midwives. 2021;34(2):e188-e95.
45. Chen S, Yue W, Liu N, Han X, Yang M. The progression on the measurement instruments of maternal health literacy: A scoping review. Midwifery. 2022;109:103308.
46. Paradies Y, Ben J, Denson N, Elias A, Priest N, Pieterse A, et al. Racism as a determinant of health: A systematic review and meta-analysis. PloS one. 2015;10(9):e0138511.
47. Thackrah RD, Wood J, Thompson SC. Longitudinal follow up of early career midwives: Insights related to racism show the need for increased commitment to cultural safety in Aboriginal maternity care. International Journal of Environmental Research and Public Health. 2021;18(3).
48. Denison J, Varcoe C, Browne AJ. Aboriginal women's experiences of accessing health care when state apprehension of children is being threatened. Journal of Advanced Nursing. 2014;70(5):1105-16.
49. Laverty M, McDermott DR, Calma T. Embedding cultural safety in Australia's main health care standards. Medical Journal of Australia. 2017;207:15-6.
50. Spangaro J, Herring S, Koziol-Mclain J, Rutherford A, Frail MA, Zwi AB. 'They aren't really black fellas but they are easy to talk to': Factors which influence Australian Aboriginal women's decision to disclose intimate partner violence during pregnancy. Midwifery. 2016;41:79-88.
51. Henning Cruickshank A, Lilley TS, Radcliffe B, Nosa V, Fenwick J. Māori and Pasifika perceptions of their local maternity care in Logan, Australia. Women and Birth. 2019;32(3):e359-e65.
52. Kildea S, Gao Y, Hickey S, Kruske S, Nelson C, Blackman R, et al. Reducing preterm birth amongst Aboriginal and Torres Strait Islander babies: A prospective cohort study, Brisbane, Australia. EClinicalMedicine. 2019;12:43-51.
53. Simpson N, Wepa D, Bria K. Improving antenatal engagement for Aboriginal women in Australia: A scoping review. Midwifery. 2020;91:102825.
54. Berra A, Kamo S, Mohnot S, Rosselot J, Suire A, Stafford J, et al. The association between antenatal maternal self-reported substance use, maternal characteristics, and obstetrical variables. Journal of Addiction Medicine. 2019;13(6):464-9.
55. Nawabi F, Alayli A, Krebs F, Lorenz L, Shukri A, Bau AM, et al. Health literacy among pregnant women in a lifestyle intervention trial: Protocol for an explorative study on the role of health literacy in the perinatal health service setting. British Medical Journal Open. 2021;11(7):e047377.
56. Allen J, Kildea S, Hartz DL, Tracy M, Tracy S. The motivation and capacity to go 'above and beyond': Qualitative analysis of free-text survey responses in the M@NGO randomised controlled trial of caseload midwifery. Midwifery. 2017;50:148-56.

For peer review only - http://bmjopen.bmj.com/site/about/guidelines.xhtml

Figure 1 Study selection

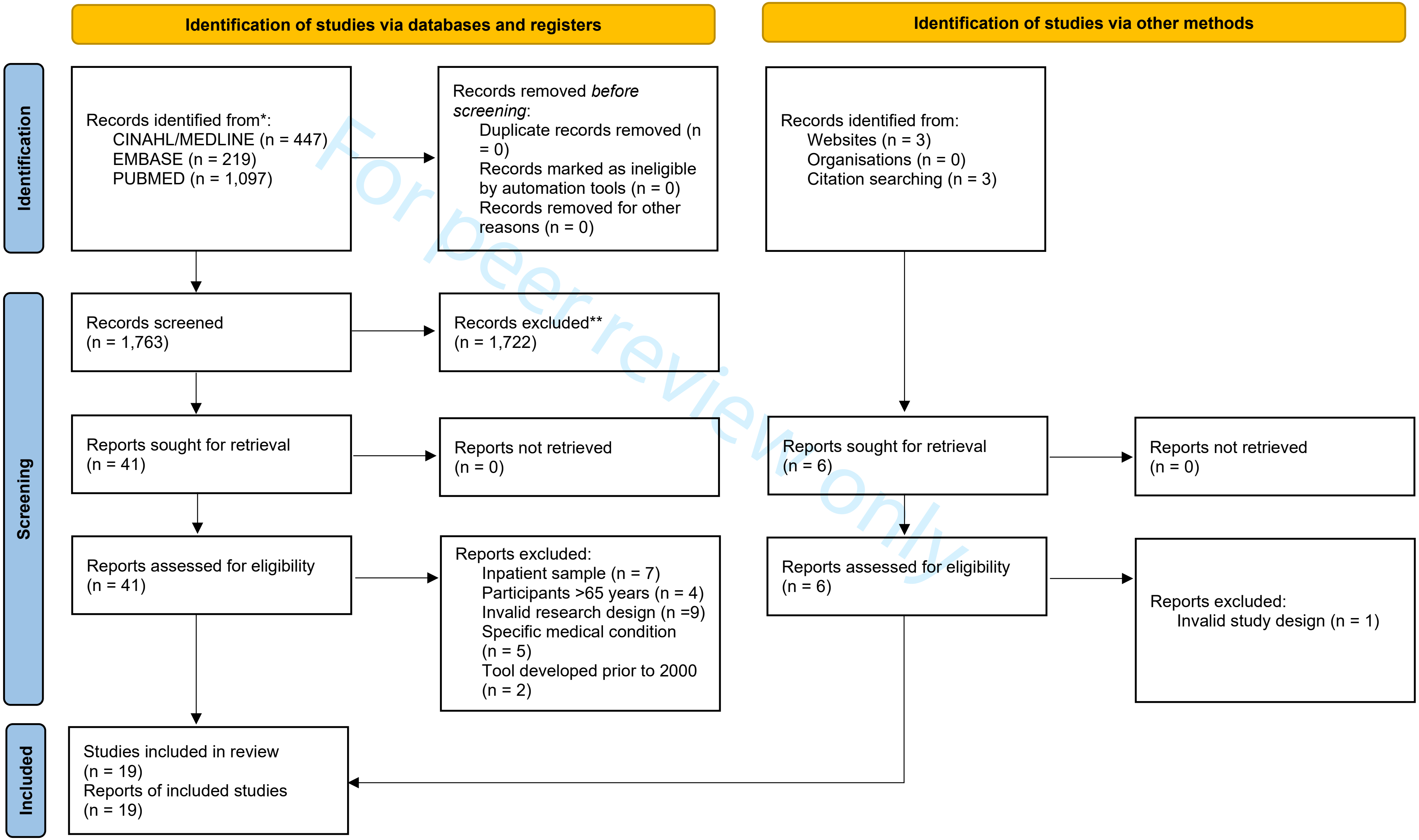

For peer review only

For peer review only

PRISMA 2020 Checklist

Section and Topic	Item #	Checklist item	Location where item is reported
**TITLE**			
Title	1	Identify the report as a systematic review.	Title page
**ABSTRACT**			
Abstract	2	See the PRISMA 2020 for Abstracts checklist.	Abstract
**INTRODUCTION**			
Rationale	3	Describe the rationale for the review in the context of existing knowledge.	Pages 1-2
Objectives	4	Provide an explicit statement of the objective(s) or question(s) the review addresses.	Page 3
**METHODS**			
Eligibility criteria	5	Specify the inclusion and exclusion criteria for the review and how studies were grouped for the syntheses.	Page 4
Information sources	6	Specify all databases, registers, websites, organisations, reference lists and other sources searched or consulted to identify studies. Specify the date when each source was last searched or consulted.	Page 4
Search strategy	7	Present the full search strategies for all databases, registers and websites, including any filters and limits used.	Page 4
Selection process	8	Specify the methods used to decide whether a study met the inclusion criteria of the review, including how many reviewers screened each record and each report retrieved, whether they worked independently, and if applicable, details of automation tools used in the process.	Pages 4-5
Data collection process	9	Specify the methods used to collect data from reports, including how many reviewers collected data from each report, whether they worked independently, any processes for obtaining or confirming data from study investigators, and if applicable, details of automation tools used in the process.	Page 4
Data items	10a	List and define all outcomes for which data were sought. Specify whether all results that were compatible with each outcome domain in each study were sought (e.g. for all measures, time points, analyses), and if not, the methods used to decide which results to collect.	Page 3
	10b	List and define all other variables for which data were sought (e.g. participant and intervention characteristics, funding sources). Describe any assumptions made about any missing or unclear information.	n/a
Study risk of bias assessment	11	Specify the methods used to assess risk of bias in the included studies, including details of the tool(s) used, how many reviewers assessed each study and whether they worked independently, and if applicable, details of automation tools used in the process.	Page 5
Effect measures	12	Specify for each outcome the effect measure(s) (e.g. risk ratio, mean difference) used in the synthesis or presentation of results.	n/a
Synthesis methods	13a	Describe the processes used to decide which studies were eligible for each synthesis (e.g. tabulating the study intervention characteristics and comparing against the planned groups for each synthesis (item #5)).	n/a
	13b	Describe any methods required to prepare the data for presentation or synthesis, such as handling of missing summary statistics, or data conversions.	n/a
	13c	Describe any methods used to tabulate or visually display results of individual studies and syntheses.	n/a
	13d	Describe any methods used to synthesize results and provide a rationale for the choice(s). If meta-analysis was performed, describe the model(s), method(s) to identify the presence and extent of statistical heterogeneity, and software package(s) used.	n/a
	13e	Describe any methods used to explore possible causes of heterogeneity among study results (e.g. subgroup analysis, meta-regression).	n/a
	13f	Describe any sensitivity analyses conducted to assess robustness of the synthesized results.	n/a
Reporting bias assessment	14	Describe any methods used to assess risk of bias due to missing results in a synthesis (arising from reporting biases).	n/a
Certainty	15	Describe any methods used to assess certainty (or confidence) in the body of evidence for an outcome.	n/a

For peer review only - http://bmjopen.bmj.com/site/about/guidelines.xhtml

PRISMA 2020 Checklist

Section and Topic	Item #	Checklist item	Location where item is reported
assessment			
**RESULTS**			
Study selection	16a	Describe the results of the search and selection process, from the number of records identified in the search to the number of studies included in the review, ideally using a flow diagram.	Page 5<br>Figure 1
	16b	Cite studies that might appear to meet the inclusion criteria, but which were excluded, and explain why they were excluded.	Figure 1
Study characteristics	17	Cite each included study and present its characteristics.	Page 5-6<br>Table 1
Risk of bias in studies	18	Present assessments of risk of bias for each included study.	Page 10<br>Table 1
Results of individual studies	19	For all outcomes, present, for each study: (a) summary statistics for each group (where appropriate) and (b) an effect estimate and its precision (e.g. confidence/credible interval), ideally using structured tables or plots.	Page 10-13
Results of syntheses	20a	For each synthesis, briefly summarise the characteristics and risk of bias among contributing studies.	n/a
	20b	Present results of all statistical syntheses conducted. If meta-analysis was done, present for each the summary estimate and its precision (e.g. confidence/credible interval) and measures of statistical heterogeneity. If comparing groups, describe the direction of the effect.	n/a
	20c	Present results of all investigations of possible causes of heterogeneity among study results.	n/a
	20d	Present results of all sensitivity analyses conducted to assess the robustness of the synthesized results.	n/a
Reporting biases	21	Present assessments of risk of bias due to missing results (arising from reporting biases) for each synthesis assessed.	n/a
Certainty of evidence	22	Present assessments of certainty (or confidence) in the body of evidence for each outcome assessed.	n/a
**DISCUSSION**			
Discussion	23a	Provide a general interpretation of the results in the context of other evidence.	Page 17-19
	23b	Discuss any limitations of the evidence included in the review.	
	23c	Discuss any limitations of the review processes used.	Page 20
	23d	Discuss implications of the results for practice, policy, and future research.	Page 20
**OTHER INFORMATION**			
Registration and protocol	24a	Provide registration information for the review, including register name and registration number, or state that the review was not registered.	Abstract<br>Page 3
	24b	Indicate where the review protocol can be accessed, or state that a protocol was not prepared.	Abstract<br>Page 3
	24c	Describe and explain any amendments to information provided at registration or in the protocol.	
Support	25	Describe sources of financial or non-financial support for the review, and the role of the funders or sponsors in the review.	Page 20
Competing interests	26	Declare any competing interests of review authors.	Page 20
Availability of	27	Report which of the following are publicly available and where they can be found: template data collection forms; data extracted from included	

For peer review only
For peer review only - http://bmjopen.bmj.com/site/about/guidelines.xhtml

PRISMA 2020 Checklist

Section and Topic	Item #	Checklist item	Location where item is reported
data, code and other materials		studies; data used for all analyses; analytic code; any other materials used in the review.	

From: Page MJ, McKenzie JE, Bossuyt PM, Boutron I, Hoffmann TC, Mulrow CD, et al. The PRISMA 2020 statement: an updated guideline for reporting systematic reviews. BMJ 2021;372:n71. doi: 10.1136/bmj.n71

For more information, visit: http://www.prisma-statement.org/

For peer review only

BMJ Open

Health engagement: A systematic review of tools modifiable for use with vulnerable pregnant women

Journal:	*BMJ Open*
Manuscript ID	bmjopen-2022-065720.R1
Article Type:	Original research
Date Submitted by the Author:	24-Jan-2023
Complete List of Authors:	Allen, Jyai; Griffith University, School of Nursing and Midwifery; Monash University School of Public Health and Preventive Medicine, Creedy, Debra; Griffith University, School of Nursing and Midwifery Mills, Kyly; Griffith University Griffith Health, School of Health Sciences and Social Work Gamble, Jenny ; Coventry University Faculty of Health and Life Sciences, School of Nursing, Midwifery and Health
**Primary Subject Heading**:	Public health
Secondary Subject Heading:	Communication, Nursing, Obstetrics and gynaecology, Patient-centred medicine
Keywords:	PRIMARY CARE, PUBLIC HEALTH, SOCIAL MEDICINE, Patient Participation, Systematic Review, Maternal medicine < OBSTETRICS

SCHOLARONE™
Manuscripts

I, the Submitting Author has the right to grant and does grant on behalf of all authors of the Work (as defined in the below author licence), an exclusive licence and/or a non-exclusive licence for contributions from authors who are: i) UK Crown employees; ii) where BMJ has agreed a CC-BY licence shall apply, and/or iii) in accordance with the terms applicable for US Federal Government officers or employees acting as part of their official duties; on a worldwide, perpetual, irrevocable, royalty-free basis to BMJ Publishing Group Ltd ("BMJ") its licensees and where the relevant Journal is co-owned by BMJ to the co-owners of the Journal, to publish the Work in this journal and any other BMJ products and to exploit all rights, as set out in our licence.

The Submitting Author accepts and understands that any supply made under these terms is made by BMJ to the Submitting Author unless you are acting as an employee on behalf of your employer or a postgraduate student of an affiliated institution which is paying any applicable article publishing charge ("APC") for Open Access articles. Where the Submitting Author wishes to make the Work available on an Open Access basis (and intends to pay the relevant APC), the terms of reuse of such Open Access shall be governed by a Creative Commons licence – details of these licences and which Creative Commons licence will apply to this Work are set out in our licence referred to above.

Other than as permitted in any relevant BMJ Author's Self Archiving Policies, I confirm this Work has not been accepted for publication elsewhere, is not being considered for publication elsewhere and does not duplicate material already published. I confirm all authors consent to publication of this Work and authorise the granting of this licence.

For peer review only

SHE systematic review

Title

Health engagement: A systematic review of tools modifiable for use with vulnerable pregnant women

Authors

Jyai Danielle Nicole **Allen**[1,2], Debra K. **Creedy**[1], Kyly **Mills**[3], Jenny **Gamble**[4, *].

Affiliations

[1] Transforming Maternity Care Collaborative, School of Nursing and Midwifery, Griffith University, 68 University Drive, Meadowbrook, Queensland, 4131, Australia. @transformingMCC

[2] School of Public Health and Preventative Medicine, Monash University, 553 St Kilda Road, Melbourne, Victoria, 3004, Australia.

[3] School of Health Sciences and Social Work, Griffith University, 1 Parklands Drive Southport, Queensland, 4215, Australia.

[4] School of Nursing, Midwifery and Health, Coventry University, Priory Street, Coventry, CV1 5FB, United Kingdom.

Corresponding author

*Jenny Gamble, School of Nursing, Midwifery and Health, Coventry University, Priory Street, Coventry, CV1 5FB, United Kingdom.

Email: ad7603@coventry.ac.uk

MeSH keywords

Maternity care; Midwifery; Patient participation; Systematic review; Surveys and questionnaires

Word count 5500

For peer review only

SHE systematic review

Abstract

Objective: to examine available health engagement tools suitable to, or modifiable for, vulnerable pregnant populations.

Design: systematic review

Eligibility criteria: original studies of tool development and validation related to health engagement, with abstract available in English, published between 2000-2022, sampling people receiving outpatient healthcare including pregnant women.

Data sources: CINAHL Complete, Medline, EMBASE, and PubMed were searched in April 2022.

Risk of bias: study quality was independently assessed by two reviewers using an adapted COSMIN risk of bias quality appraisal checklist. Tools were also mapped against the Synergistic Health Engagement model which centres on women's buy-in to maternity care.

Included studies: nineteen studies were included from Canada, Germany, Italy, Netherlands, Sweden, the United Kingdom, and the United States. Four tools were used with pregnant populations, two tools with vulnerable non-pregnant populations, six tools measured patient-provider relationship, four measured patient activation, and three tools measured both relationship and activation.

Results: tools that measured engagement in maternity care assessed some of the following constructs: communication or information sharing, woman-centred care, health guidance, shared decision-making, sufficient time, availability, provider attributes, discriminatory or respectful care. None of the maternity engagement tools assessed the key construct of buy-in. While non-maternity health engagement tools measured some elements of buy-in (self-care, feeling hopeful about treatment), other elements (disclosing risks to healthcare providers and acting on health advice) which are significant for vulnerable populations were rarely measured.

Conclusions and implications: Health engagement is hypothesised as the mechanism by which midwifery-led care reduces the risk of perinatal morbidity for vulnerable women. To test this hypothesis, a new assessment tool is required that addresses all the relevant constructs of the Synergistic Health Engagement model, developed for, and psychometrically assessed in the target group.

Registration: PROSPERO database (CRD42020214102).

Strength and limitations:

- Health engagement in maternity care is an emerging concept and terms used in this review may not have captured novel or less-used terms
- Study quality was independently assessed by two reviewers using a quality appraisal checklist
- The theoretical model used to synthesise results was missing two constructs relevant to vulnerable populations: health literacy and cultural safety.

For peer review only - http://bmjopen.bmj.com/site/about/guidelines.xhtml

SHE systematic review

Introduction

Health engagement is the willingness and ability of people to actively participate in their health and navigate healthcare systems (1). This definition includes two related but separate constructs in relation to health and healthcare: activation and health literacy (2). Activation includes the motivation and confidence to autonomously manage one's health and healthcare; and has been associated with positive health outcomes in several populations with chronic conditions (3). Health literacy is the extent to which individuals can "obtain, process and understand basic health information"(4), which impacts their behaviours and outcomes (5). In psychology and related disciplines, the concept of engagement is often measured by therapeutic alliance between patient/client and provider. Therapeutic alliance refers to a collaborative and emotional bond between patient/client and provider that predicts better psychological outcomes with greater certainty than specific interventions (6),

A recent systematic review demonstrated that therapeutic alliance is rarely measured in nursing, most commonly in mental health nursing (8 papers)(7). A preliminary literature search by the authors found it has never been measured in midwifery, and there has been only one qualitative study of therapeutic alliance in the context of maternity care (8). Several systematic reviews demonstrate that engagement interventions can improve patients' knowledge and experience, use of health service, health behaviour and health outcomes (9). However, there are few theoretical frameworks available to inform research about patient engagement, alongside a lack of validated and specific measurement tools (10). Furthermore, the extent to which such tools are suitable for vulnerable pregnant populations is under-researched.

Vulnerable pregnant women

The term *vulnerable women* encapsulates those women most at risk for poorer maternal health outcomes. In Australia, these populations include adolescent mothers; Aboriginal and Torres Strait Islander mothers; culturally and linguistically diverse mothers; mothers from lower socio-economic backgrounds and mothers who reside in regional and remote areas. Vulnerable women are more likely to have socially determined risk factors which independently predict poor pregnancy outcomes like low birth weight and/or preterm birth (11). These factors have been systematically researched and include depression (12), intimate partner violence (13), housing instability (14), environmental chemical exposure including tobacco (15), illicit drug use (16), inadequate or excessive gestational weight gain (17),

For peer review only - http://bmjopen.bmj.com/site/about/guidelines.xhtml

genital tract infection (18), and late or inadequate use of antenatal care (19). Importantly, the aforementioned factors are modifiable through access to, and engagement with, high quality maternity care.

In order to achieve optimal benefits, maternity care models need to do more than enable pregnant women to just *turn up* to their maternity appointments; the system needs to facilitate *buy-in* (20). People buy-in when they make “emotional investment and commitment” to healthcare because they are enabled and believe it is “worthwhile and beneficial”(21). People demonstrate buy-in when they participate in health promoting behaviours (e.g. attend for care, have screening tests); and participate in self-care activities including nutrition and exercise, while minimising harm (e.g., reducing or ceasing substance use).(3) Pregnant women buy-in because they hope and believe that this will improve the health and well-being of themselves and their babies (11). Furthermore, patient buy-in is predicated on trust in health providers’ advice and guidance (22).

There is a clear need to measure health engagement to better understand and address the poorer maternal health outcomes of pregnant women from vulnerable groups. However, we were unable to locate any reviews of health engagement tools for use with pregnant women. Many available health engagement tools have been developed with inpatients who have chronic health conditions, which may not translate to healthy pregnant women receiving outpatient primary care. This gap highlights the need to critically review existing tools to determine their applicability for use with vulnerable pregnant women.

Objectives

This review aimed to (1) describe the nature of health engagement tools used in outpatient populations with or without chronic health conditions; (2) evaluate the reliability and validity of those tools; and (3) use a health engagement framework to evaluate which tools, or components of tools, could be modified for use with vulnerable pregnant populations.

Methods

The systematic review protocol was registered with PROSPERO (number CRD42020214102).

For peer review only

For peer review only - http://bmjopen.bmj.com/site/about/guidelines.xhtml

SHE systematic review

Theoretical framework

This review was informed by elements of Synergistic Health Engagement (SHE) - an empirical model based on integrated findings from mixed methods research with pregnant adolescents accessing caseload midwifery (11). Caseload midwifery provides continuity through one-to-one, relationship-based care during pregnancy, birth, and until the first six weeks (23). According to the SHE model, the first three constructs create the conditions for *buy-in to maternity care* (the fourth construct) which modifies *socially determined predictors of perinatal morbidity* (the fifth construct). The five constructs and their elements are listed below:

1) **Optimal model of care**: continuity of midwifery carer, with 24-hour telephone availability, with community-based, flexible visits
2) **Midwife's attributes:** the skills and personal qualities that enable her to be empowering, trustworthy, and empathetic.
3) **Philosophy / best practice:** the midwife's use of health promotion, woman-centred care, shared decision-making.
4) **Woman's buy-in:** having hope and belief that maternity care will be beneficial, disclosing risk factors, trusting and acting on health advice, and engaging in self-care.
5) **Modifiable social risks for perinatal morbidity**: inadequate antenatal care; smoking, alcohol, or drug use; poor mental health; family violence; inappropriate gestational weight gain; and genito-urinary infection (20).

The SHE model explains the mechanism by which caseload midwifery reduces the likelihood of poor pregnancy outcomes for vulnerable women (20). Therefore, SHE was a suitable framework to assess the construct of tools to measure health engagement in maternity care. Application of the SHE model informed the synthesis of items included in the tools, and the interpretation of findings.

Eligibility criteria

Inclusion criteria: primary research with study populations of adolescents or adults; and used structured tools to assess healthcare engagement or a related construct (including patient activation, patient-professional relationship).

Exclusion criteria: primary research population inpatients, children or elderly (mean age 65 years or older); engagement not related to receiving healthcare (e.g., technology, research, policy, patient safety); participation not related to healthcare (e.g., work or community

For peer review only - http://bmjopen.bmj.com/site/about/guidelines.xhtml

participation); ineligible study design (pilot studies, intervention studies, qualitative studies, case studies, systematic reviews); earlier iterations of tools which had since been revised or validation of translated tools; shared decision-making tools; tools focussed on a specific health condition.

Search strategy and information sources

Table 1 details the search that was conducted in April 2022.

Table 1. Search strategy

Databases	Search terms	Filters
Cumulative Index of Nursing and Allied Health Literature (CINAHL) Complete	patient activation OR patient empowerment OR patient engagement OR patient involvement OR patient participation	Publication type - questionnaire/scale
Medline and EMBASE	(patient activation OR patient empowerment OR patient engagement OR patient involvement OR patient participation)<br>AND<br>(tool OR index OR measure OR scale OR instrument OR questionnaire)	Date range (2000 – 2022)<br>Peer-reviewed<br>Age groups 13 – 44 years
PubMed	Medical Subject Heading (MeSH) term *patient participation* AND MeSH major heading *surveys and questionnaires.*	

Selection and data collection process

We reviewed articles identified by these searches and relevant references found within those articles. When we identified multiple eligible papers from the same study, we included the main paper reporting validity and reliability of the tool. For every eligible study, two reviewers extracted data on study design, setting, sample size, population type, age range, research design, name of tool, and psychometric properties of the tool.

Study risk of bias assessment

To our knowledge, there is no suitable tool for assessment of health engagement tools for this population. Therefore, two reviewers independently assessed the quality of included studies

For peer review only - http://bmjopen.bmj.com/site/about/guidelines.xhtml

using a study-specific tool. We adapted the COSMIN Risk of Bias checklist and updated criteria for measurement properties (24). Our eleven items for measurement properties included: clear purpose, theoretical constructs, content validity, pilot test, structural validity, internal consistency, reliability, as well as criterion, convergent and predictive validity. Each item was scored according to 1 = adequate or 0 = doubtful/inadequate. Scores were summed with scores of 9 - 11 = high quality; 5 – 8 = moderate quality, and < 5 = poor quality. A third reviewer was consulted if disagreements occurred, and a majority decision was reached. We investigated the theoretical basis of included tools and compared identified constructs with elements of the SHE model to evaluate utility of tools for use with vulnerable pregnant populations. Ethical approval was not required for this review.

Patient and public involvement

None

Results

Study selection

Figure 1 reports the number of studies obtained, screened, and reviewed through databases and other methods. The database search strategy resulted in two authors reading 41 full-text articles to further determine eligibility and resolve any disagreement about inclusion by discussion and consensus. A third reviewer was consulted if disagreements occurred, and a majority decision was reached. Systematic reviews were screened, and where indicated, read in full to search for additional tools, but systematic reviews were not themselves included in this study. Figure 1 indicates reasons for excluding articles from the systematic review following close reading. The authors were aware of three relevant tools that met the eligibility criteria but were not located through the search of databases. A website search for these three tools was conducted, they were located and included in the review.

Study characteristics

Nineteen tools related to health engagement from 19 studies published between 2000 – 2022 were included. Table 2 summarises the key characteristics of the 19 tools included in the review. Studies were conducted in Canada (25-28), Germany (29, 30), Italy (31, 32), the Netherlands(33), Sweden (34), the United Kingdom (35, 36), and the United States (37-43). Participants were men and women who were 11 years or older; mean age of participants in each of the included studies was <65 years of age. Four tools were tested with the pregnant

For peer review only - http://bmjopen.bmj.com/site/about/guidelines.xhtml

population (25-27, 43). Of these, two tools were co-designed with vulnerable pregnant women (e.g., refugee, socially disadvantaged)(25, 27), and one study adapted a pre-existing tool (Interpersonal Care Processes) to suit a pregnant population (Prenatal Interpersonal Care Processes)(43).

Fifteen studies included non-pregnant participants. Of these, two studies focussed on engagement with mental health services (34, 35), one focussed on engagement with child protective services (42), and one solely recruited adolescents (37). The remaining 11 studies, focussed on health engagement in the general adult population with or without chronic conditions, sampled through online or mailed surveys (30, 40), in hospital outpatient clinics (29, 31, 32, 34, 38, 39, 41), and general practitioner surgeries (33, 36). Three studies used the following conceptual frameworks to design their health engagement instruments; the *Patient Health Engagement Model* (PHE-Scale) (32), a conceptual model of outcomes influenced by primary care (PCOQ-24)(36), and therapeutic alliance (KAS-R).(38)

Risk of bias in studies

All tools were valid and reliable with at least adequate quality rated on the 11-point scale. Twelve tools were assessed as high quality (scores 9-11), seven tools were assessed as fair (scores 5-8). The difference in quality related to the extent and adequacy of testing (Table 2).

Results of individual studies

The scales, subscales, and items of the 19 included tools were reviewed and mapped against the SHE model constructs (optimal model of care, midwife attributes, best practice principles buy-in) and related elements. No tool measured all components, and one tool (32) measured none of them.

For peer review only

Table 2: Characteristics and quality of included tools

Year Country	Tool and aim	Population	Study design	Quality score
**Tools designed for pregnant population accessing maternity care**				
2017 (25) *Canada*	**Mothers Autonomy in Decision Making (MADM) scale.** To develop and test a scale to assess women's autonomy and role in decision making in pregnancy	1,672 women with a single provider during pregnancy	Literature review to generate new items and adapt previous items, modification through survey, focus groups and expert panel review. Four working groups refined content with immigrant and refugee women, formerly incarcerated women, women facing socio-economic barriers, and urban/rural settings. Pilot testing with target population with minor rewording.	**9**
2017 (27) *Canada*	**Mothers on Respect (MoR) index.** To assess women's experiences with maternity care, including disrespect and discrimination			
2014 (26) *Canada*	**Quality of Prenatal Care Questionnaire (QPCQ).** Develop an instrument to measure the quality of prenatal care	422 postpartum women (preliminary) 422 postpartum women (final)	Preliminary tool developed following interviews with pregnant women and health providers, review of prenatal care guidelines, assessment of content validity and rating of importance of items. Preliminary testing and exploratory factor analysis to generate and test final version with separate sample of participants.	**10**
2004 (43) *US*	**Prenatal Interpersonal Processes of Care (PIPC).** Develop a reliable and valid multidimensional measure of prenatal interpersonal processes of care for use with ethnically diverse women	363 African American, Latino, Caucasian pregnant women (low-income)	Adaptation of the Interpersonal Processes of Care tool. Focus groups were used to test face validity of items prior to telephone survey with the 30-item tool.	**10**
**Tools designed for non-pregnant population accessing healthcare**				
2021 (28) *Canada*	**CADICEE.** To co-construct a tool for measuring degree of partnership between patients and healthcare providers.	206 patients and 38 relatives present during consultation	Patients co-constructed tool. Qualitative analysis of patient experience performed by research team. Patient research advisory group involved in entire construction of tool process. Tool tested for construct and convergent validity and internal consistency.	**7**
2019 (31) *Italy*	**Patient-Professional Interaction Questionnaire (PPIQ)**. To investigate how patient evaluate provision of patient-centre care by healthcare professionals and psychometrically test a questionnaire.	1139 inpatients and outpatients	A self-assessment of professionals' provision of PCC was adapted into a patient-rated form. The questionnaire structure, reliability, susceptibility to social desirability, and associations with other variables were tested.	**8**
2019 (30) *Germany*	**Patient Enablement (PEN-13).** To develop and psychometrically test a German-language survey instrument that measures patient enablement generically and in greater detail than previous instruments	354 adults registered with the integrated care system	Analysis of key concepts from the literature by a multidisciplinary team informed item development. Construct and structural validity, and internal consistency were tested.	**9**
2018 (36) *UK*	**Primary Care Outcomes Questionnaire (PCOQ).** To test the PCOQ which aims to capture a broad range of outcomes relevant to primary care.	602 adult general practitioner patients with and without chronic conditions	Questionnaire developed through use of a conceptual model, interviews with patients and clinicians, expert review through Delphi study, and refinement of tool through cognitive interviews prior to psychometric testing.	**10**

For peer review only - http://bmjopen.bmj.com/site/about/guidelines.xhtml

2015 (32) *Italy*	**Patient Health Engagement Scale (PHE-Scale).** To evaluate the psychometric properties of the PHE-scale and to evaluate the association between PHE-scale scores and concurrent measures.	382 adults with chronic disease	Items based on previous conceptualization of patient engagement (the PHE-model). Items based on a systematic analysis of the literature and an extensive qualitative study. Tool was pilot-tested and validated using confirmatory factor analysis.	**10**
2015 (40) *US*	**Altarum Consumer Engagement (ACE) measure.** To assess an individual's engagement in health and healthcare decisions.	2,079 adults through online survey	Item generation through literature review, piloting and refinement of items. Testing of tool through web-based survey	**9**
2014 (37) *US*	**Youth Engagement with Health Services (YEHS!).** To create and validate a survey instrument designed to measure youth engagement with health services	354 ethnically diverse high school students, school health centres	Item generation through literature review including existing validated measures, expert opinion, and interviews with adolescents. Instrument pilot tested and refined.	**8**
2010 (29) *Germany*	**Questionnaire on the Quality of Physician-Patient Interaction (QQPPI).** To assess the quality of physician-patient interactions.	147 adults and 19 physicians in outpatient clinic	Item generation from exploratory in-depth interviews with 20 patients. Literature review used to screen for additional items. An expert panel reviewed the final 14-item questionnaire.	**10**
2008 (34) *Sweden*	**Health Promotion Intervention Questionnaire (HPIQ).** To measure patients' subjective experiences of health promotion interventions in mental health services.	135 adults who accessed a mental health service	Item generation informed by qualitative research; cross-section survey used to pilot test tool.	**7**
2008 (38) *US*	**Kim Alliance Scale – Revised (KAS-R).** To measure quality of the therapeutic alliance from patient's perspective, including patient empowerment.	601 participants from two outpatient clinics	Conceptual framework developed through literature review and qualitative research. Original 48-item KAS tool piloted as paper-based survey and refined to 30-items. KAS tool further refined to 16-items through exploratory and validation studies.	**10**
2005 (41) *US*	**Patient Activation Measure (PAM-13).** To conceptualize and measure "activation" in patients and consumers	1,515 adults with or without a chronic condition	Mixed methods study including national expert consensus and patient focus groups to define and identify domains. Pilot testing and refinement prior to national survey.	**10**
2005 (42) *US*	**Client Engagement in Child Protective Services.** To develop and test a multidimensional measure of client engagement in child welfare services.	287 primary caregivers with an open child protective services case	Five dimensions of engagement based on literature review, interviews with child welfare workers and clients. Three expert panels provided feedback on items, validity, and format. Construct validity and internal consistency were tested.	**8**
2004 (35) *UK*	**Engagement measure (self-report version).** To develop a reliable self-report measure of engagement based on Hall, Meaden, Smith, & Jones 2001 observer-rated measure of engagement	25 unemployed adults accessing outreach mental health services	Tool developed based on an observer-rated measure of engagement. The *process* of developing the altered self-report measure of engagement not provided. Construct validity, including factor analysis were not assessed.	**6**
2004 (33) *Holland*	**Patient-Doctor Relationship Questionnaire (PDRQ-9).** To develop and validate a questionnaire to assess the patient-doctor relationship	110 general practice patients, 55 epilepsy clinic patients	The HAQ of Luborsky basis for item creation. Pilot testing (n = 8) resulted in the additional item. Factor analysis, test re-test reliability and internal consistency were undertaken.	**6**
2001 (39) *US*	**Facilitation of Patient Involvement (FPI) scale** To measure the degree to which patients perceive their physicians actively facilitate or encourage involvement in their own healthcare.	236 adults (pilot) 338 adults (survey)	Item generation through literature review and revision based on expert feedback. Pilot study of 9-item scale using 6-point Likert responses.	**10**

For peer review only - http://bmjopen.bmj.com/site/about/guidelines.xhtml

SHE systematic review

Health engagement tools for pregnant women

Four tools were designed to measure health engagement in maternity care. Both the MADM tool (25) and MoR index (27) measured shared decision-making. In addition, the MoR index (27) measured providers attributes associated with (dis)respectful care. While the MoR index (27) measured the unwillingness of pregnant women to ask questions or share concerns with their provider, it did not specifically measure disclosure of risks, which is a significant element of buy-in. The 2014 QPCQ (26) included six subscales: 1) information sharing, 2) anticipatory guidance, 3) sufficient time, 4) approachability, 5) availability, 6) support and respect. The QPCQ (26) subscales mapped against optimal model of care, provider attributes, and best practice principles including health promotion; but did not measure buy-in. The 2004 PIPC (43) included three dimensions (subscales): 1) communication (elicitation of, and responsiveness to patient; explanations of processes of care; empowerment/self-care); 2) patient-centred decision making; and 3) interpersonal style (friendliness and courteousness; respectfulness/emotional support; lack of perceived discrimination). The PIPC (43) measured provider attributes including being perceived as discriminatory, empowering, and empathetic. The PIPC (43) measured elements of best practice including woman-centred care and shared decision-making. While the PIPC (43) was developed with an ethnically diverse, low-income sample of pregnant women, it does not measure buy-in.

Health engagement tools for vulnerable people

Two tools were designed for people who were vulnerable due to age (adolescents), or engagement with child protective services. The Youth Engagement in Health Services (YEHS!)(37) survey measured adolescent health engagement across four components: 1) receipt of anticipatory health guidance, 2) experience of care, 3) health access literacy, and 4) health self-efficacy. Health topics included in the anticipatory guidance component are relevant to health promotion during pregnancy; and could be easily adapted in future tool development. Yatchmenoff (2005) developed a 19-item client-centred health engagement model with four factors: 1) buy-in, 2) receptivity, 3) working relationship, and 4) mistrust (42). The factors, and items, in this tool are relevant to buy-in and receptivity of vulnerable pregnant populations and the midwife-woman relationship.

General health activation tools

The Patient Activation Measure (PAM-13) (41) was the first tool to define the construct of patient activation, and is the most widely used measure of patient engagement. The 13 items focus on the patient's perceived knowledge, confidence and ability to manage their health

For peer review only - http://bmjopen.bmj.com/site/about/guidelines.xhtml

condition (41). While commonly used, the tool has limited generalisability to vulnerable populations because of readability for people with low health literacy (44). Furthermore, because activation is constructed solely in terms of *confidence* and *ability*, this obfuscates significant barriers to health engagement that occur through social determinants of health and poor patient-provider relationships.

The Patient Enablement (PEN-13) questionnaire (German version) focusses specifically on measuring skills patients without chronic conditions had to manage their health care (30). Like the PAM-13 (41), this tool measures an aspect of patient engagement without the context of the patient-provider relationship, which limits usefulness for modification for a maternity tool.

The Patient Health Engagement (PHE) scale (32) focusses on patients who receive a health diagnosis, and measures items that occur prior to, and predict, the individual's ability to engage in health care. For example, *When I think about my disease: I feel blackout, I am in alarm, I am aware, I feel positive* (32). For both reasons, the PHE scale (32) is not relevant to maternity health engagement.

The Altarum Consumer Engagement (ACE) measure (40) included 20-items evenly divided across four subscales: 1) commitment, 2) informed choice, 3) navigation, and 4) ownership. The ACE (40) measures individuals' confidence to navigate the healthcare system and take responsibility for their health. Only one item refers to interaction with a health professional (i.e., *I feel comfortable talking to my doctor about my health*) (40).

Patient-provider relationship tools

Six tools focussed on health engagement through the lens of the patient-provider relationship. The Kim Alliance Scale Revised (KAS-R) (38) was developed to measure *therapeutic alliance* in healthcare. The KAS-R (38) included 16 items evenly divided across four subscales: 1) collaboration, 2) integration, 3) empowerment, and 4) communication. Items which measured mutual goals and respect are useful to health engagement for pregnant women. The wording of some items (e.g., *I am allowed in the decision-making process*) (38) reflects that this tool was developed over 15-years ago, prior to significant progress in the area of shared decision-making. More recently published tools, like the MADM tool (25) have more appropriate wording and description of decision-making in maternity care.

The Patient Doctor Relationship Questionnaire (PDRQ-9) (33) contained nine short and general, and potentially vague, statements about satisfaction with relationship with the

For peer review only - http://bmjopen.bmj.com/site/about/guidelines.xhtml

primary care provider (e.g., *My primary care provider understands me*). In comparison, the Questionnaire on Quality of Physician-Patient Interactions (QQPPI) (29) contained 14 statements which were more specific (e.g., *The physician seemed genuinely interested in my problems*). Likewise, the Facilitation of Patient Involvement (FPI) scale (39) asked patients to rate how often their physician does nine things. For example, *My doctor explains all the treatment options to me so that I can make an informed choice.*(39) The Patient-Professional Interaction Questionnaire (PPIQ) (31) included 16 items, of which several measured whether the professional was interested in (e.g. *what I know about my disease/prognosis, what I want from care*). The Health Promotion Intervention Questionnaire (HPIQ) (34) measures patient experiences of health promotion as received through their relationship with a key worker. The HPIQ (34) consists of 19-items across four factors: Alliance, Empowerment, Educational Support, and Practical Support. The items are commonly superficially worded (e.g., *Key worker treats me in a friendly way and often smiles*) which does not measure the therapeutic nature of the patient-provider relationship.

Comprehensive health engagement tools

Three tools measured both patient-provider relationship and components of patient activation. The CADICEE tool (28) comprehensively addresses the complex components of health engagement using 24 items across seven dimensions: 1) relationship of **C**onfidence or trust between patients and health care providers, 2) patient **A**utonomy, 3) patient participation in **D**ecisions related to care, 4) shared **I**nformation on patient health status and care, 5) patient personal **C**ontext, 6) **E**mpathy, and 7) recognition of **E**xpertise (28).

The PCOQ-24 (36) has 24-items across four dimensions: 1) health and wellbeing, 2) health knowledge and self-care, 3) confidence in health provision, and 4) confidence in health plan. Items ask about the skills and attributes of *the doctors or nurses you usually see* as well as patient knowledge and ability to self-manage their health condition (36).

The Engagement Measure (client version) (35) has six key areas: 1) appointment keeping, 2) client-keyworker relationship, 3) communication/openness with key workers, 4) usefulness of treatment, 5) involvement with treatment, and 6) taking medication. The professional's attributes were not described (*e.g., How well do you get on with _______?*) which makes items so general that their value is limited.

For peer review only - http://bmjopen.bmj.com/site/about/guidelines.xhtml

Synthesis of Results

The results from all studies were synthesised through mapping against SHE model constructs of model of care; health professional's skills and attributes; best practice; and buy-in.

Model of care

The availability and flexibility of the health professional to meet the individual's needs, were largely measured in terms of having sufficient time and being contactable. For example, the PIPC item: *How often did providers give you enough time to say what you thought was important?*(43) The QPCQ(26) differentiated between being able to contact *someone* versus *my provider* when concerns arose. Four general health engagement tools (QQPPI(29), PPIQ(31), PDRQ-9 (33), YEHS! (37) included items about having *enough time* with a health professional. Only one general tool (PDRQ-9(33)) measured accessibility: *I find my primary care provider easily accessible*.

Health professional's skills and attributes

Most tools include subscales or items that measured whether the individual perceived their provider as empowering, trustworthy, or empathetic; or provider actions that could be interpreted to demonstrate these qualities. Of the four maternity tools, two tools included the terms 'doctor or midwife' and asked respondents to indicate which healthcare provider their responses referred to (25, 27). Whereas the QPCQ referred generally to a *prenatal care provider* (26) and the PIPC measured experiences about *doctors, nurses and other providers* (43).

Empowering

An empowering health professional refers to someone who uses communication skills to effectively share information with pregnant women. Any items that were specifically about how the health professional facilitated shared decision-making were mapped against that element of best practice. Two maternity tools measured attributes related to whether the health professional was empowering. For example, the QPCQ item: *I fully understood the reasons for blood work and other tests that my prenatal care provider(s) ordered for me* (26) and the PIPC item: *How often did providers tell you what they were doing as they gave you a physical examination?*(43)

For peer review only - http://bmjopen.bmj.com/site/about/guidelines.xhtml

Most non-maternity tools included at least one item that measured whether the health professional acted in ways that were empowering (QQPPI (29), PPIQ (31), PCOQ (36), CADICEE (28), PAM-13 (41), KAS-R (38), FPI (39), PEN-13 (30), YEHS! (37)). The KAS-R (38) included an Empowerment subscale with items such as: *I have an active partnership with my provider*. Whereas the FPI (39) included a negatively worded item: *My doctor discourages my questions.*

Trustworthy

Three maternity tools (QPCQ (26), PIPC (43), MoR (27)) measured outcomes related to perceptions of the health professional being trustworthy, including perceived discrimination, support, and respect. However, only two tools specifically measured racism. For example, the PIPC item: *How often did you feel discriminated against because of your race or ethnicity?* (43). Whereas the MoR included multiple items related to disrespectful care including *When I had my baby I felt that I was treated poorly by my (midwife, doctor): Because of my race, ethnicity, cultural background or language* (27).

A third of the non-maternity tools (YEHS! (37), CECPS (42), CADICEE (28), PCOQ (36), PDRQ-9 (33)) measured patient perceptions of patient-provided relationship in terms of trust. For example, the YEHS! item: *I have a safe and trusting relationship with at least one doctor or healthcare provider* (37). Whereas the PCOQ-24 (36) included a more generic question regarding doctors and nurses in the general practice: *You can trust them.* This item does not speak to a patient-provider relationship, nor continuity of care with a specific provider, so it of limited utility.

Empathetic

Both the QPCQ (26) and PIPC (43) have sub-scales related to empathy including demonstrating interest in the woman as a person and communicating emotional support. For example, the QPCQ item: *My prenatal care provider was interested in how my pregnancy was affecting my life* (26) and the PIPC item: *How often did providers help you feel less worried about your pregnancy?*(43)

About half of the general tools (YEHS! (37), QQPPI (29), CADICEE (28), PPIQ (31), KAS-R (38), HPIQ (34)) measured patient-provider relationship in terms of empathy. Similarly, to the PIPC item, a QQPPI item measured the ability of the provider to reassure the patient: *The physician did all he/she could to put me at ease* (29). Whereas some tools more explicitly

For peer review only - http://bmjopen.bmj.com/site/about/guidelines.xhtml

measured the emotional element of the patient-provider relationship. For example, the PPIQ item: *He/she was able to put him/herself in "my shoes"*(31).

Best practice principles

In the SHE model, health promotion, woman-centred care and shared decision-making were captured as a construct titled *philosophical commitments*, in other words, best practice principles.

Health promotion

Health promotion in maternity care includes discussion and recommendations around diet, exercise, emotional health, interpersonal relationships, social support, harm minimisation for smoking, drugs and alcohol (20). Two included maternity tools measured constructs directly related to promotion of healthy behaviours. A QPCQ subscale measured whether women were provided with education about components of health including diet, exercise, alcohol, emotional health; for example: *I was given adequate information about depression in pregnancy* (26). In contrast, a PIPC sub-scale asked more broadly about whether pregnant women had received lifestyle advice (43). The PIPC also included an item about the health professional's role in promoting the woman to engage in healthy behaviours: *How often did providers tell you what you could do to take care of yourself and your pregnancy at home?* (43)

Few non-maternity tools included items around health promotion. However, both PAM-13 (41) and ACE (40) included several items around patient responsibility in self-care and healthy behaviours. Whereas the HPIQ, designed for participants with mental health concerns, includes the item: *My key worker informs me about what I need in order to feel better* (34). The YEHS! was the most relevant because it provided a list of specific educational topics beyond diet and exercise, relevant to vulnerable populations:

> *In the **last 12 months**, did a doctor or other health care provider talk with you about the following: weight, healthy eating or diet, physical activity or exercise, your emotions or moods, how you deal with stress, sleep, sexual risk reduction (STDs)* (37).

Woman-centred / individualised care

Most maternity tools measured whether the individual felt care was individualised to their specific values, preferences, and desires (QPCQ (26), MoR (27), PIPC (43)). For example, the QPCQ item: *I was supported by my prenatal care provider(s) in doing what I felt was*

For peer review only - http://bmjopen.bmj.com/site/about/guidelines.xhtml

right for me (26). Only three non-maternity tools assessed whether the provider communicated interest in what the patient knew and wanted from their care (QQPPI (29), CADICEE (28), PPIQ (31)). For example, the CADICEE tool measured a further element of individualisation around patient expertise about themselves and their health: *Did you feel that this professional recognized your expertise and took it into account?*(28).

Shared decision-making

Shared decision-making was measured in all four maternity tools. The most comprehensive measures were included in the MADM tool (25) and MoR index (27) which were codesigned with vulnerable pregnant women. Example items include: *My doctor or midwife explained the advantages and disadvantages of the maternity care options* (MADM (25)) and *Overall while making decision during my pregnancy I felt coerced into accepting the options my (midwife, doctor) recommended* (MoR (27)). About half of the non-maternity tools included items that measured shared decision-making (QQPPI (29),CADICEE (28), PPIQ (31), KAS-R(38), FPI (39), HPIQ (34)). For example, the QQPPI item: *The physician and I made treatment decisions together* (29).

Buy-in

Buy-in was not measured by the included maternity tools, whereas each element (self-care, hope/belief, disclosure of risk, and accepting help) was measured by some of the non-maternity tools.

Self-care

Several non-maternity tools (PAM-13 (41), ACE (40), PCOQ (36), PEN-13 (30)) measured the individuals' confidence and willingness to manage their health including knowing when and how to access help, following through on plans and goals, maintaining a healthy lifestyle, and preventing deterioration of their health. For example, the PAM-13 item *I am confident that I can take actions that will help prevent or minimise some symptoms or problems associated with my health condition* (41). These items were generally worded around a diagnosed health condition, which does not translate easily to pregnancy. The PCOQ-24 (36) included a more general question around self-care and health: *Thinking about your level of knowledge, how much do you know how best to look after yourself and stay healthy.*

Hope and belief

An individual's mindset, their interpretation of whether healthcare is "worth it" or not, is a motivating factor in health engagement. This element was measured by a third of the non-

For peer review only - http://bmjopen.bmj.com/site/about/guidelines.xhtml

maternity tools (CECPS (42), PCOQ (36), PPIQ (31), EM (35), HPIQ (34)), commonly in a single item. The CECPS (42) included five items that measured mutual goals and optimism for a better future through engagement with child protective services. For example, *Working with ______ has given me more hope about how my life is going to go in the future* (42).

Disclosure

The element of disclosure of risk factors (e.g., family violence) was not specifically measured by any of the 19 included tools. Of the four maternity tools, only the MoR index (27) included three items regarding whether women perceived they were held back from asking questions or discussing concerns. About half of the non-maternity tools (YEHS!, CECPS (42), QQPPI (29), CADICEE (28), EM, PAM-13 (41), PEN-13 (30)) included items related to disclosure. For example, whether individuals could share problems or concerns without being directly asked by the provider (*I have no difficulty in telling my doctor about my concerns or fears, even if he or she does not address them directly* – PEN-13 (30)). Whereas the CECPS includes an item about non-disclosure (*Anything I say they're going to turn it around and make me look bad*) (42).

Accepting help, referral, treatment

Acceptance of help, including agreeing to referrals and following up with tests, community resources and support from other providers was not measured. Some maternity tools measured whether information was provided about screening tests (43). Providing an explanation is empowering, and may increase the likelihood of women doing the test, but it does not measure buy-in. Agreeing to treatment, going along with treatment, and wanting help were measured by non-maternity tools. A third of the non-maternity tools (YEHS! (37), CECPS (42), PCOQ (36), EM (35), PAM-13 (41)) measured willingness to accept or act on help offered by the care provider. For example, *When I make a plan with a doctor or other health care provider, I can follow through on the plan at home* in the YEHS! (37) The PCOQ-24 include items with greater specificity around follow through, for example: *How much of your doctors or nurses advice are you following on: your medication or treatment; leading a healthy lifestyle (36).*

Discussion

This is the first systematic review to evaluate the characteristics, quality, and sufficiency of tools to measure health engagement in vulnerable pregnant populations. There is Level 1 evidence that midwifery-led care delivers a 24% reduction in preterm birth (23). For women

For peer review only - http://bmjopen.bmj.com/site/about/guidelines.xhtml

at highest risk, like First Nations, there is emerging evidence that the rate of reduction is 38% (45). No other clinical intervention delivers a benefit of this magnitude to prevent preterm birth. Yet how midwifery-led care works to reduce preterm birth is not clear. The SHE model hypothesises that midwifery-led care works, through the mechanism of health engagement, to impact modifiable, social predictors of preterm birth (20). This hypothesis requires testing through measuring health engagement in vulnerable women who access midwifery-led versus others models of care. The results of this systematic review indicate that a tool needs to be developed which is psychometrically validated to measure health engagement for vulnerable pregnant women. Twelve tools were high quality and validated for use with generally healthy adolescents and adults in high-income healthcare settings. The SHE framework was useful to map constructs to potential scales, subscales, and items. Buy-in was not measured in the included maternity tools, and only partially measured in some of the general patient engagement tools. Specifically, disclosure and accepting help were rarely measured.

The review was limited to studies with abstracts available in English published from 2000 onwards. There may be studies in different languages, or published prior to 2000, that were not located or excluded from review. While we did review tools used with other populations, our focus on concepts of care relevant to pregnant women may have been limiting. However, registration of the review protocol confirmed the relative lack of attention health engagement generally and with pregnant women specifically. The inclusion of tools that were psychometrically tested with individuals with chronic disease (e.g., mental health, diabetes) were a poor fit for vulnerable pregnant women who are generally physically healthy. The breadth of included tools may therefore be considered a limitation. Although, application of the SHE model as a framework for the review ensured that a range of relevant concepts were considered, two elements (health literacy and cultural safety) were identified as missing. Inclusion of these elements from the outset may have influenced the search terms and outcomes. Furthermore, the SHE model has been tested only with adolescent mothers.

Health literacy

Elements of health literacy were broadly measured including how well participants could understand instructions or explanations provided to them by care providers (KAS-R (38), QQPPI (29)); or how users sought and used information (ACE (40)). Interestingly, the provider's knowledge and skills around maternal health literacy were not measured in the included maternity engagement tools. A national survey reported that most Australian midwives have not received education about health literacy, do not formally assess it, and

For peer review only - http://bmjopen.bmj.com/site/about/guidelines.xhtml

either never or only sometimes use techniques to facilitate it (46). There are currently no rapid measures of maternal health literacy available (47).

Cultural safety

Racism undermines equitable access to services and predicts poor maternal and perinatal outcomes (48). Internationally, First Nations women experience racism, prejudice and discrimination, and lack of respect for cultural practices in maternity care (49, 50). Indeed, social risk factors are exacerbated by health care that is perceived as racist or culturally unsafe; because it contributes to reduced adherence to health advice or complete disengagement from services (51). Women who do not wish to attend, or are prevented from attending, antenatal care are less likely to receive advice, screening and reassurance about the progress of pregnancy. Beyond attendance, cultural safety is central to women's decision to disclose risk factors, like domestic violence, to their care providers (52). Perceived lack of cultural safety through hospital-based, fragmented care is a barrier to attendance, whereas culturally safe, midwifery continuity of care in the community is facilitative (53). A recent study of the impact of culturally safe maternity care, concluded that synergistic engagement is the mechanism by which these innovative services effectively improve maternal and perinatal outcomes for First Nations women in Australia (54). However, in the absence of a tool validated to measure SHE constructs, this explanation remains theoretical.

Antenatal attendance

The term *engagement* is commonly used as a synonym for *attendance* in the research literature. For example, a recent scoping review on improving antenatal engagement for Aboriginal women used measures of non-attendance, less attendance, and late attendance (55). While antenatal attendance is important, without *buy-in* to maternity care and resultant modification of social risk factors, its' efficacy is limited. The ability to determine appropriate medical and/or psychosocial intervention is predicated on women feeling safe to disclose risks to their maternity care providers (56). Future studies of maternity care engagement should include specific measures that can assess not only the quantity of engagement (attendance) but quality.

Relationship-based care

Testing of health promoting interventions, delivered in the absence of relationship-based care, has been proposed to improve perinatal outcomes (57). While health promotion is an important part of quality maternity care, the provision of motivational counselling around

For peer review only - http://bmjopen.bmj.com/site/about/guidelines.xhtml

social risk factors, does not necessarily translate into women acting on advice. A trusting, provider-woman relationship is foundational to pregnant women's willingness and ability to engage (20). Women randomised to receive midwifery continuity, versus fragmented care, characterised their midwife as *empowering* and *'endorphic'* (makes me feel safe, loved, relaxed); compared to simply informative, competent, kind (58). We recommend interventions designed to promote healthy behaviours during pregnancy are embedded in relationship-based models of care to bolster their efficacy.

Implications for practice and future research

Maternal health engagement is crucial to improving perinatal outcomes for vulnerable groups. Models of maternity care can be designed to enhance or hinder engagement. Therefore, routine measurement of health engagement is an important activity to monitor and improve the quality of maternity care for vulnerable pregnant women. While 12 tools were deemed to be high quality, no single tool addressed all relevant constructs. A new assessment tool is required that addresses all the relevant constructs, developed for, and psychometrically assessed in the target group.

For peer review only

For peer review only - http://bmjopen.bmj.com/site/about/guidelines.xhtml

Figure 1 Legend: Study selection

Competing interests' statement: The authors declare no competing interests.

Funding statement: This research received no specific grant from any funding agency public, commercial or not-for-profit.

Author contributions: JDNA: conceived and designed the analysis, collected the data, analysed the results, wrote the paper. **DKC:** conceived and design the analysis, performed quality appraisal of included articles, revised the paper. **KM:** performed quality appraisal of included articles, revised the paper. **JG:** conceived and designed the analysis, revised the paper.

Ethical approval statement: This study did not involve animal or human participants; therefore, ethical approval was not required.

Data sharing statement: Data sharing not applicable as no datasets generated and/or analysed for this study.

For peer review only

For peer review only - http://bmjopen.bmj.com/site/about/guidelines.xhtml

SHE systematic review

References

1. Friis K, Lasgaard M, Osborne RH, Maindal HT. Gaps in understanding health and engagement with healthcare providers across common long-term conditions: A population survey of health literacy in 29 473 Danish citizens. British Medical Journal Open. 2016;6(1):e009627.
2. Smith SG, Curtis LM, Wardle J, von Wagner C, Wolf MS. Skill set or mind set? Associations between health literacy, patient activation and health. PloS one. 2013;8:e74373.
3. Graffigna G, Barello S, Bonanomi A. The role of Patient Health Engagement Model (PHE-model) in affecting patient activation and medication adherence: A structural equation model. PloS one. 2017;12:e0179865.
4. Ratzan SC. Health literacy: Communication for the public good. Health Promotion International. 2001;16(2):207-14.
5. Kilfoyle KA, Vitko M, O'Conor R, Bailey SC. Health literacy and women's reproductive health: A systematic review. Journal of Women's Health. 2016;25(12):1237-55.
6. Martin DJ, Garske JP, Davis MK. Relation of the therapeutic alliance with outcome and other variables: A meta-analytic review. Journal of Consulting and Clinical Psychology. 2000;68(3):438-50.
7. Hartley S, Raphael J, Lovell K, Berry K. Effective nurse-patient relationships in mental health care: A systematic review of interventions to improve the therapeutic alliance. International Journal of Nursing Studies. 2020;102:103490.
8. Doherty ME. Therapeutic alliance: A concept for the childbearing season. J Perinat Educ. 2009;18(3):39-47.
9. Coulter A, Ellins J. Effectiveness of strategies for informing, educating, and involving patients. British Medical Journal 2007;335(7609):24-7.
10. Manafo E, Petermann L, Mason-Lai P, Vandall-Walker V. Patient engagement in Canada: A scoping review of the 'how' and 'what' of patient engagement in health research. Health Research Policy and Systems. 2018;16(1):5.
11. Allen JDN. (How) does the way maternity care is provided affect the health and well-being of young women and their babies? A mixed methods research project [thesis]. Brisbane, Australia: Australian Catholic University; 2015.
12. Accortt EE, Cheadle AC, Dunkel Schetter C. Prenatal depression and adverse birth outcomes: An updated systematic review. Journal of Maternal and Child Health. 2015;19(6):1306-37.
13. Nesari M, Olson JK, Vandermeer B, Slater L, Olson DM. Does a maternal history of abuse before pregnancy affect pregnancy outcomes? A systematic review with meta-analysis. BMC pregnancy and childbirth. 2018;18(1):404.
14. DiTosto JD, Holder K, Soyemi E, Beestrum M, Yee LM. Housing instability and adverse perinatal outcomes: A systematic review. American Journal of Obstetrics & Gynecology MFM. 2021;3(6):100477.
15. Porpora MG, Piacenti I, Scaramuzzino S, Masciullo L, Rech F, Benedetti Panici P. Environmental contaminants exposure and preterm birth: A systematic review. Toxics. 2019;7(1):11.
16. Dos Santos JF, de Melo Bastos Cavalcante C, Barbosa FT, Gitaí DLG, Duzzioni M, Tilelli CQ, et al. Maternal, fetal and neonatal consequences associated with the use of crack cocaine during the gestational period: A systematic review and meta-analysis. Archives of Gynecology and Obstetrics. 2018;298(3):487-503.
17. Goldstein RF, Abell SK, Ranasinha S, Misso M, Boyle JA, Black MH, et al. Association of gestational weight gain with maternal and infant outcomes: A systematic review and meta-analysis. Journal of the American Medical Association. 2017;317(21):2207-25.
18. Sangkomkamhang US, Lumbiganon P, Prasertcharoensuk W, Laopaiboon M. Antenatal lower genital tract infection screening and treatment programs for preventing preterm delivery. Cochrane Database of Systematic Reviews. 2015;2015(2):Cd006178.
19. Feijen-de Jong EI, Jansen DE, Baarveld F, van der Schans CP, Schellevis FG, Reijneveld SA. Determinants of late and/or inadequate use of prenatal healthcare in high-income countries: A systematic review. European Journal of Public Health. 2012;22(6):904-13.

For peer review only - http://bmjopen.bmj.com/site/about/guidelines.xhtml

20. Allen J, Kildea S, Stapleton H. How optimal caseload midwifery can modify predictors for preterm birth in young women: Integrated findings from a mixed methods study. Midwifery. 2016;41:30-8.
21. Gopalan G, Goldstein L, Klingenstein K, Sicher C, Blake C, McKay MM. Engaging families into child mental health treatment: Updates and special considerations. Journal of the Canadian Academy of Child and Adolescent Psychiatry 2010;19(3):182-96.
22. Becker ER, Roblin DW. Translating primary care practice climate into patient activation: The role of patient trust in physician. Medical Care. 2008;46(8):795-805.
23. Sandall J, Soltani H, Gates S, Shennan A, Devane D. Midwife-led continuity models versus other models of care for childbearing women. The Cochrane database of systematic reviews. 2016;4:Cd004667.
24. Prinsen CAC, Mokkink LB, Bouter LM, Alonso J, Patrick DL, de Vet HCW, et al. COSMIN guideline for systematic reviews of patient-reported outcome measures. Qual Life Res. 2018;27(5):1147-57.
25. Vedam S, Stoll K, Martin K, Rubashkin N, Partridge S, Thordarson D, et al. The Mother's Autonomy in Decision Making (MADM) scale: Patient-led development and psychometric testing of a new instrument to evaluate experience of maternity care. PloS one. 2017;12(2):e0171804.
26. Heaman MI, Sword WA, Akhtar-Danesh N, Bradford A, Tough S, Janssen PA, et al. Quality of prenatal care questionnaire: Instrument development and testing. BMC pregnancy and childbirth. 2014;14:188.
27. Vedam S, Stoll K, Rubashkin Nea. The Mothers on Respect (MOR) index: Measuring quality, safety, and human rights in childbirth. SSM - Population Health. 2017;3:201-10.
28. Pomey MP, Clavel N, Normandin L, Del Grande C, Philip Ghadiri D, Fernandez-McAuley I, et al. Assessing and promoting partnership between patients and health-care professionals: Co-construction of the CADICEE tool for patients and their relatives. Health Expectations. 2021;24(4):1230-41.
29. Bieber C, Müller KG, Nicolai J, Hartmann M, Eich W. How does your doctor talk with you? Preliminary validation of a brief patient self-report questionnaire on the quality of physician-patient interaction. Journal of Clinical Psychology in Medical Settings. 2010;17(2):125-36.
30. Siegel A, Ehmann AT, Meyer I, Gröne O, Niebling W, Martus P, et al. PEN-13: a new generic 13-item questionnaire for measuring patient enablement (German version). International Journal of Environmental Research and Public Health. 2019;16(23):4867.
31. Casu G, Gremigni P, Sommaruga M. The Patient-Professional Interaction Questionnaire (PPIQ) to assess patient centered care from the patient's perspective. Patient Education and Counseling. 2019;102(1):126-33.
32. Graffigna G, Barello S, Bonanomi A, Lozza E. Measuring patient engagement: Development and psychometric properties of the Patient Health Engagement (PHE) Scale. Frontiers in Psychology. 2015;6.
33. Van der Feltz-Cornelis CM, Van Oppen P, Van Marwijk HW, De Beurs E, Van Dyck R. A patient-doctor relationship questionnaire (PDRQ-9) in primary care: Development and psychometric evaluation. General Hospital Psychiatry. 2004;26(2):115-20.
34. Svedberg P, Arvidsson B, Svensson B, Hansson L. Psychometric characteristics of a self-report questionnaire (HPIQ) focusing on health promotion interventions in mental health services. International Journal of Mental Health Nursing. 2008;17(3):171-9.
35. Gillespie M, Smith J, Meaden A, Jones C, Wane J. Clients' engagement with assertive outreach services: A comparison of client and staff perceptions of engagement and its impact on later engagement. Journal of Mental Health. 2004;13(5):439-52.
36. Murphy M, Hollinghurst S, Cowlishaw S, Salisbury C. Primary Care Outcomes Questionnaire: Psychometric testing of a new instrument. British Journal of General Practice. 2018:bjgp18X695765.

For peer review only

37. Sebastian RA, Ramos MM, Stumbo S, McGrath J, Fairbrother G. Measuring youth health engagement: Development of the youth engagement with health services survey. Journal of Adolescent Health. 2014;55(3):334-40.
38. Kim SC, Kim S, Boren D. The quality of therapeutic alliance between patient and provider predicts general satisfaction. Military Medicine. 2008;173(1):85-90.
39. Martin LR, Robin Di Matteo M, Lepper HS. Facilitation of Patient Involvement in Care: Development and Validation of a Scale. Behavioral medicine (Washington, DC). 2001;27(3):111-20.
40. Duke CC, Lynch WD, Smith B, Winstanley J. Validity of a new patient engagement measure: The Altarum Consumer Engagement (ACE) measure. Patient. 2015;8(6):559-68.
41. Hibbard JH, Stockard J, Mahoney ER, Tusler M. Development of the Patient Activation Measure (PAM): Conceptualizing and measuring activation in patients and consumers. Health Services Research 2005;39(4p1):1005-26.
42. Yatchmenoff DK. Measuring client engagement from the client's perspective in nonvoluntary child protective services. Research on Social Work Practice. 2005;15(2):84-96.
43. Wong ST, Korenbrot CC, Stewart AL. Consumer assessment of the quality of interpersonal processes of prenatal care among ethnically diverse low-income women: Development of a new measure. Womens Health Issues. 2004;14.
44. Wolf MS, Smith SG, Pandit AU, Condon DM, Curtis LM, Griffith J, et al. Development and validation of the Consumer Health Activation Index. Medical Decision Making. 2018;38(3):334-43.
45. Kildea S, Gao Y, Hickey S, Nelson C, Kruske S, Carson A, et al. Effect of a Birthing on Country service redesign on maternal and neonatal health outcomes for First Nations Australians: a prospective, non-randomised, interventional trial. The Lancet Global Health. 2021;9(5):e651-e9.
46. Creedy DK, Gamble J, Boorman R, Allen J. Midwives' self-reported knowledge and skills to assess and promote maternal health literacy: A national cross-sectional survey. Women and birth : journal of the Australian College of Midwives. 2021;34(2):e188-e95.
47. Chen S, Yue W, Liu N, Han X, Yang M. The progression on the measurement instruments of maternal health literacy: A scoping review. Midwifery. 2022;109:103308.
48. Paradies Y, Ben J, Denson N, Elias A, Priest N, Pieterse A, et al. Racism as a determinant of health: A systematic review and meta-analysis. PloS one. 2015;10(9):e0138511.
49. Thackrah RD, Wood J, Thompson SC. Longitudinal follow up of early career midwives: Insights related to racism show the need for increased commitment to cultural safety in Aboriginal maternity care. International Journal of Environmental Research and Public Health. 2021;18(3).
50. Denison J, Varcoe C, Browne AJ. Aboriginal women's experiences of accessing health care when state apprehension of children is being threatened. Journal of Advanced Nursing. 2014;70(5):1105-16.
51. Laverty M, McDermott DR, Calma T. Embedding cultural safety in Australia's main health care standards. Medical Journal of Australia. 2017;207:15-6.
52. Spangaro J, Herring S, Koziol-Mclain J, Rutherford A, Frail MA, Zwi AB. 'They aren't really black fellas but they are easy to talk to': Factors which influence Australian Aboriginal women's decision to disclose intimate partner violence during pregnancy. Midwifery. 2016;41:79-88.
53. Henning Cruickshank A, Lilley TS, Radcliffe B, Nosa V, Fenwick J. Māori and Pasifika perceptions of their local maternity care in Logan, Australia. Women and Birth. 2019;32(3):e359-e65.
54. Kildea S, Gao Y, Hickey S, Kruske S, Nelson C, Blackman R, et al. Reducing preterm birth amongst Aboriginal and Torres Strait Islander babies: A prospective cohort study, Brisbane, Australia. eClinicalMedicine. 2019;12:43-51.
55. Simpson N, Wepa D, Bria K. Improving antenatal engagement for Aboriginal women in Australia: A scoping review. Midwifery. 2020;91:102825.
56. Berra A, Kamo S, Mohnot S, Rosselot J, Suire A, Stafford J, et al. The association between antenatal maternal self-reported substance use, maternal characteristics, and obstetrical variables. Journal of Addiction Medicine. 2019;13(6):464-9.

For peer review only - http://bmjopen.bmj.com/site/about/guidelines.xhtml

57. Nawabi F, Alayli A, Krebs F, Lorenz L, Shukri A, Bau AM, et al. Health literacy among pregnant women in a lifestyle intervention trial: Protocol for an explorative study on the role of health literacy in the perinatal health service setting. British Medical Journal Open. 2021;11(7):e047377.
58. Allen J, Kildea S, Hartz DL, Tracy M, Tracy S. The motivation and capacity to go 'above and beyond': Qualitative analysis of free-text survey responses in the M@NGO randomised controlled trial of caseload midwifery. Midwifery. 2017;50:148-56.

For peer review only

For peer review only - http://bmjopen.bmj.com/site/about/guidelines.xhtml

Figure 1 Study selection

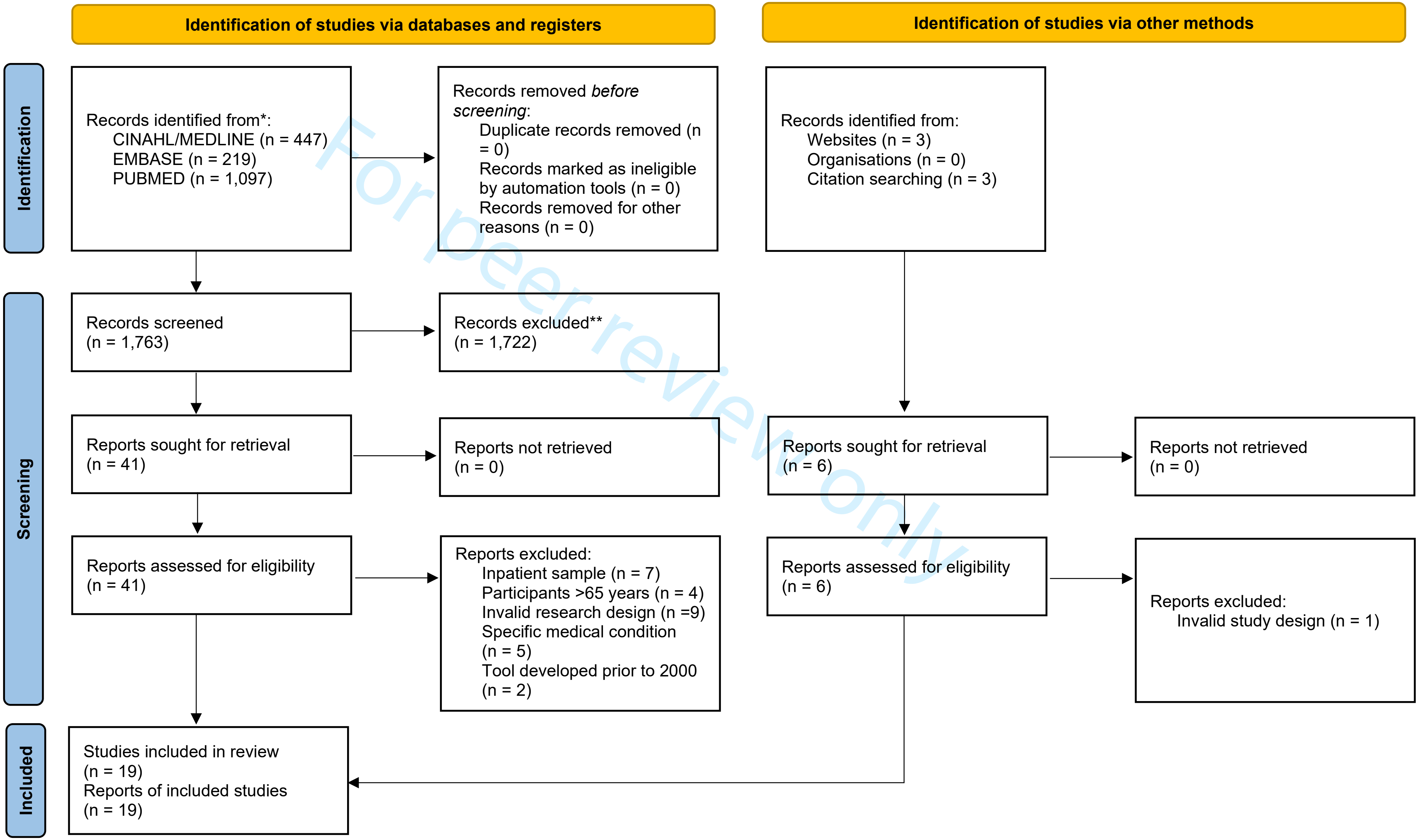

For peer review only

For peer review only

PRISMA 2020 Checklist

Section and Topic	Item #	Checklist item	Location where item is reported
**TITLE**			
Title	1	Identify the report as a systematic review.	Title page
**ABSTRACT**			
Abstract	2	See the PRISMA 2020 for Abstracts checklist.	Abstract
**INTRODUCTION**			
Rationale	3	Describe the rationale for the review in the context of existing knowledge.	Pages 1-2
Objectives	4	Provide an explicit statement of the objective(s) or question(s) the review addresses.	Page 3
**METHODS**			
Eligibility criteria	5	Specify the inclusion and exclusion criteria for the review and how studies were grouped for the syntheses.	Page 4
Information sources	6	Specify all databases, registers, websites, organisations, reference lists and other sources searched or consulted to identify studies. Specify the date when each source was last searched or consulted.	Page 4
Search strategy	7	Present the full search strategies for all databases, registers and websites, including any filters and limits used.	Page 4
Selection process	8	Specify the methods used to decide whether a study met the inclusion criteria of the review, including how many reviewers screened each record and each report retrieved, whether they worked independently, and if applicable, details of automation tools used in the process.	Pages 4-5
Data collection process	9	Specify the methods used to collect data from reports, including how many reviewers collected data from each report, whether they worked independently, any processes for obtaining or confirming data from study investigators, and if applicable, details of automation tools used in the process.	Page 4
Data items	10a	List and define all outcomes for which data were sought. Specify whether all results that were compatible with each outcome domain in each study were sought (e.g. for all measures, time points, analyses), and if not, the methods used to decide which results to collect.	Page 3
	10b	List and define all other variables for which data were sought (e.g. participant and intervention characteristics, funding sources). Describe any assumptions made about any missing or unclear information.	n/a
Study risk of bias assessment	11	Specify the methods used to assess risk of bias in the included studies, including details of the tool(s) used, how many reviewers assessed each study and whether they worked independently, and if applicable, details of automation tools used in the process.	Page 5
Effect measures	12	Specify for each outcome the effect measure(s) (e.g. risk ratio, mean difference) used in the synthesis or presentation of results.	n/a
Synthesis methods	13a	Describe the processes used to decide which studies were eligible for each synthesis (e.g. tabulating the study intervention characteristics and comparing against the planned groups for each synthesis (item #5)).	n/a
	13b	Describe any methods required to prepare the data for presentation or synthesis, such as handling of missing summary statistics, or data conversions.	n/a
	13c	Describe any methods used to tabulate or visually display results of individual studies and syntheses.	n/a
	13d	Describe any methods used to synthesize results and provide a rationale for the choice(s). If meta-analysis was performed, describe the model(s), method(s) to identify the presence and extent of statistical heterogeneity, and software package(s) used.	n/a
	13e	Describe any methods used to explore possible causes of heterogeneity among study results (e.g. subgroup analysis, meta-regression).	n/a
	13f	Describe any sensitivity analyses conducted to assess robustness of the synthesized results.	n/a
Reporting bias assessment	14	Describe any methods used to assess risk of bias due to missing results in a synthesis (arising from reporting biases).	n/a
Certainty	15	Describe any methods used to assess certainty (or confidence) in the body of evidence for an outcome.	n/a

For peer review only - http://bmjopen.bmj.com/site/about/guidelines.xhtml

PRISMA 2020 Checklist

Section and Topic	Item #	Checklist item	Location where item is reported
assessment			
**RESULTS**			
Study selection	16a	Describe the results of the search and selection process, from the number of records identified in the search to the number of studies included in the review, ideally using a flow diagram.	Page 5<br>Figure 1
	16b	Cite studies that might appear to meet the inclusion criteria, but which were excluded, and explain why they were excluded.	Figure 1
Study characteristics	17	Cite each included study and present its characteristics.	Page 5-6<br>Table 1
Risk of bias in studies	18	Present assessments of risk of bias for each included study.	Page 10<br>Table 1
Results of individual studies	19	For all outcomes, present, for each study: (a) summary statistics for each group (where appropriate) and (b) an effect estimate and its precision (e.g. confidence/credible interval), ideally using structured tables or plots.	Page 10-13
Results of syntheses	20a	For each synthesis, briefly summarise the characteristics and risk of bias among contributing studies.	n/a
	20b	Present results of all statistical syntheses conducted. If meta-analysis was done, present for each the summary estimate and its precision (e.g. confidence/credible interval) and measures of statistical heterogeneity. If comparing groups, describe the direction of the effect.	n/a
	20c	Present results of all investigations of possible causes of heterogeneity among study results.	n/a
	20d	Present results of all sensitivity analyses conducted to assess the robustness of the synthesized results.	n/a
Reporting biases	21	Present assessments of risk of bias due to missing results (arising from reporting biases) for each synthesis assessed.	n/a
Certainty of evidence	22	Present assessments of certainty (or confidence) in the body of evidence for each outcome assessed.	n/a
**DISCUSSION**			
Discussion	23a	Provide a general interpretation of the results in the context of other evidence.	Page 17-19
	23b	Discuss any limitations of the evidence included in the review.	
	23c	Discuss any limitations of the review processes used.	Page 20
	23d	Discuss implications of the results for practice, policy, and future research.	Page 20
**OTHER INFORMATION**			
Registration and protocol	24a	Provide registration information for the review, including register name and registration number, or state that the review was not registered.	Abstract<br>Page 3
	24b	Indicate where the review protocol can be accessed, or state that a protocol was not prepared.	Abstract<br>Page 3
	24c	Describe and explain any amendments to information provided at registration or in the protocol.	
Support	25	Describe sources of financial or non-financial support for the review, and the role of the funders or sponsors in the review.	Page 20
Competing interests	26	Declare any competing interests of review authors.	Page 20
Availability of	27	Report which of the following are publicly available and where they can be found: template data collection forms; data extracted from included	

For peer review only

PRISMA 2020 Checklist

Section and Topic	Item #	Checklist item	Location where item is reported
data, code and other materials		studies; data used for all analyses; analytic code; any other materials used in the review.	

From: Page MJ, McKenzie JE, Bossuyt PM, Boutron I, Hoffmann TC, Mulrow CD, et al. The PRISMA 2020 statement: an updated guideline for reporting systematic reviews. BMJ 2021;372:n71. doi: 10.1136/bmj.n71

For more information, visit: http://www.prisma-statement.org/

For peer review only
